# Specific FAHFAs predict worsening glucose tolerance in non-diabetic relatives of people with Type 2 diabetes

**DOI:** 10.1016/j.jlr.2025.100819

**Published:** 2025-05-05

**Authors:** Ismail Syed, Ken Sluis, Pratik Aryal, Zachary Solomon, Rucha Patel, Srihari Konduri, Dionicio Siegel, Ulf Smith, Barbara B. Kahn

**Affiliations:** 1 Division of Endocrinology, Diabetes and Metabolism, Department of Medicine, Beth Israel Deaconess Medical Center and Harvard Medical School, Boston, MA, USA; 2 Skaggs School of Pharmacy and Pharmaceutical Sciences, University of California San Diego, La Jolla, CA, USA; 3 The Lundberg Laboratory for Diabetes Research, Departments of Molecular and Clinical Medicine, Institute of Medicine, Sahlgrenska Academy at the University of Gothenburg, Gothenburg, Sweden

**Keywords:** impaired glucose tolerance, Type 2 diabetes, lipid biomarkers, anti-diabetic and anti-inflammatory lipids, fatty acid hydroxy fatty acids, palmitic acid hydroxy stearic acids, palmitic acid hydroxy oleic acids, BMI, HOMA

## Abstract

There is a growing need for early biomarkers for Type 2 diabetes (T2D). Fatty-Acid-Hydroxy-Fatty-Acids (FAHFAs) are bioactive lipids with >580 regioisomers in human tissues. FAHFAs such as Palmitic Acid Hydroxy Stearic Acids (PAHSAs) are anti-diabetic and anti-inflammatory. PAHSA concentrations in human serum and adipose tissue strongly correlate with insulin sensitivity. Since PAHSAs and palmitic acid hydroxy oleic acids (PAHOAs) are among the most abundant FAHFAs in human serum, we investigated whether they predict worsening glucose tolerance in first-degree relatives of people with T2D. All participants had normal glucose tolerance (NGT) at baseline; 27 remained NGT (NGT-NGT) and 21 developed impaired glucose tolerance (NGT-IGT). In NGT-NGT, total PAHSA and PAHSA regioisomer concentrations were unchanged from baseline to follow-up, while in NGT-IGT participants, most PAHSA regioisomers decreased. The initial total PAHSAs, 5-PAHSA, and 9-PAHSA, and changes in these correlated inversely with worsening glucose tolerance. Low total PAHSA concentrations at baseline and the decrease in total PAHSAs, 5-PAHSAs, and 9-PAHSAs over time predicted IGT independent of initial BMI or %body fat, change in BMI or in %body fat, initial fasting glucose, fasting insulin, or triglyceride/HDL ratio. In contrast, baseline and follow-up total PAHOA and PAHOA regioisomer levels were higher in NGT-IGT than NGT-NGT, and some PAHOA regioisomers increased during follow-up in NGT-IGT. Higher initial total PAHOAs predicted IGT independent of the same clinical variables. Thus, lower serum PAHSAs and higher PAHOAs predict worsening glucose tolerance/IGT independent of BMI, %body fat, or change in these parameters, even in lean, relatively young people.

Type 2 diabetes (T2D) is a global epidemic with 463 million adults affected (1). T2D incidence is increasing in childhood and adolescence, causing earlier onset of complications, including cardiovascular and renal disease, which could shorten life span (2, 3). Identification of people with increased T2D risk is important so that lifestyle and medical management can be instituted early to prevent progression. The need for sensitive and early biomarkers of IGT and T2D is even more compelling with recent advances in medical therapies, which can prevent T2D and its complications (4, 5, 6).

Branched fatty acid hydroxy fatty acids (FAHFAs) are bioactive lipids which are present in mammals and plants and are evolutionarily conserved in lower organisms (7, 8). At least 84 different combinations of fatty acids and hydroxy fatty acids with branching at different carbons are present in human white adipose tissue (8, 9). FAHFAs can be synthesized in human adipocytes via transacylation by adipose triglyceride lipase (10). Many FAHFAs, but not all, have beneficial metabolic and anti-inflammatory effects (8, 9, 10, 11, 12, 13, 14, 15, 16, 17, 18, 19). One FAHFA family, palmitic acid hydroxy stearic acids (PAHSAs), has been extensively studied for its antidiabetic and anti-inflammatory effects (8, 11, 12). Administration of 5- or 9-PAHSA to insulin-resistant high-fat diet (HFD)-fed mice improves glucose tolerance and systemic and hepatic insulin sensitivity (11, 12). Some PAHSA isomers dramatically increase glucose-stimulated insulin secretion from human islets (8, 11, 13) and restore normal pulsatility of insulin secretion in islets from people with T2D (14, 15). Both 5- and 9-PAHSA delay the onset and markedly attenuate Type 1 Diabetes incidence in autoimmune non-obese diabetic mice (16). PAHSAs also directly protect pancreatic β-cells independent of immune modulation by attenuating cytokine-induced cell death, endoplasmic reticulum stress, and metabolic-stress-induced senescence (16, 17). Other FAHFA family members also augment basal metabolism and insulin sensitivity in mice on an HFD/high-sugar diet and in genetically obese mice (18, 19).

In humans, levels of specific FAHFAs are regulated in serum, plasma, and adipose tissue by age, exercise, diet, short-term overfeeding, laparoscopic sleeve gastrectomy, and in pathophysiological states (20, 21, 22, 23), PAHSA levels are low in serum and subcutaneous adipose tissue of insulin-resistant people and levels correlate highly with insulin sensitivity (8, 24). The 5-PAHSA levels are reduced in breast milk from women with obesity compared to lean women (25). Plasma levels of several FAHFAs correlate with cardiovascular biomarkers in healthy humans (23).

In this study, we investigated whether serum PAHSA or PAHOA regioisomers predict IGT or T2D. This is the first longitudinal study investigating this question. The data indicate that serum levels of specific FAHFA families and regioisomers may predict T2D risk independent of other classic risk factors.

## Materials and Methods

### Participants

We recruited 48 healthy first-degree relatives of one or more people with T2D from advertisements in newspapers and fliers in the outpatient clinic. Informed consent was obtained before enrollment. All subjects were healthy, not taking any chronic medication, non-diabetic, and had normal glucose tolerance (NGT) at initial testing (baseline). Definitions of NGT, IGT, and T2D were based on the 1997 American Diabetes Association criteria (26) for glucose values obtained after an overnight fast and a 2h oral glucose-tolerance test (OGTT), conducted with a standard loading dose of 75g. Plasma and serum samples were collected after an overnight fast. Standard techniques were used to measure plasma glucose, insulin, and serum lipids (27). Lean body mass and fat mass were assessed using dual-energy X-ray absorptiometry (DEXA) scans (Delphi A; Hologic). Clinical and metabolic characteristics are shown in Tables 1 and 2. Throughout a rolling enrollment period (3–12 years), 27 participants maintained NGT while 21 participants developed IGT. One participant was omitted because of a technical issue while measuring FAHFA levels. Six participants (one NGT-IGT and 5 NGT-NGT group) did not have follow-up serum HDL, LDL, and triglyceride levels. However, these participants were included in analyses of other parameters.

**Table 1:** Clinical and Metabolic characteristics of human participants at baseline (Initial)

Initial Characteristics	NGT to NGT (n = 27)	NGT to IGT (n = 21)	Initial Groups compared
	Mean ± SD (Range)	Mean ± SD (Range)	*P*-Value
Sex (Female/Male)	7/20	17/4	0.0001
Age	35 ± 7 (28–51)	43 ± 5 (29–50)	0.0001
BMI	25.0 ± 2.8 (20–30)	25.3 ± 3.5 (19–31)	NS
WHR	0.89 ± 0.1 (0.7–1.2)	0.86 ± 0.1 (0.69–1.0)	NS
% Body Fat	21.1 ± 7.9 (7.4–32.6)	29.5 ± 8.1 (18–44)	0.0003
Fasting Glucose (mg/dl)	85 ± 9 (70–115)	85 ± 8 (70–99)	NS
Fasting Insulin (pmol/L)	51 ± 26 (21–109)	44 ± 17 (21–79)	NS
HOMA (μU/ml/mmol/L)	1.6 ± 0.9 (0.6–3.5)	1.3 ± 0.5 (0.6–2.5)	NS
OGTT 2h value (mg/dl)	91 ± 23 (47–130)	115 ± 15 (86–137)	0.0001
Area under curve – OGTT (mg/dl)	776 ± 123 (635–1,107)	866 ± 150 (633–1,251)	0.029
Total Cholesterol (mmol/L)	4.7 ± 0.79 (2.8–6.3)	5.0 ± 1.08 (3.5–7.1)	NS
LDL (mmol/L)	2.7 ± 0.7 (1.3–4.1)	2.9 ± 1.0 (1.3–5.6)	NS
HDL (mmol/L)	1.5 ± 0.4 (0.7–2.5)	1.7 ± 0.4 (0.9–2.7)	NS
Triglycerides (mmol/L)	0.9 ± 0.4 (0.4–2.2)	1.1 ± 0.5 (0.6–2.5)	NS
Triglyceride/HDL	0.7 ± 0.4 (0.2–2.0)	0.7 ± 0.5 (0.3–2.0)	NS

All participants were nondiabetic. Blood was drawn after an overnight fast. Data are expressed as means ± SD. Differences between groups were assessed by Student's *t* test.

BMI, Body Mass Index; HDL, High Density Lipoproteins; IGT, Impaired Glucose Tolerance; LDL, Low Density Lipoproteins; NGT, Normal Glucose Tolerance; NS, Not Significant; OGTT, Oral Glucose Tolerance Test; WHR, Waist Hip Ratio.

**Table 2:** Clinical and Metabolic characteristics of human participants at follow-up

Follow-up Characteristics	NGT to NGT (n = 27)	NGT to IGT (n = 21)	Follow-up Groups Compared	Initial to Follow-up	
				NGT	IGT
	Mean ± SD (Range)	Mean ± SD (Range)	*P*-Value	*P*-Value	*P*-Value
Follow-up Duration (Years)	6.7 ± 3 (3–12)	6.9 ± 3 (3–12)	NS	n/a	n/a
Age	42 ± 10 (31–61)	50 ± 6 (38–58)	0.001	<0.0001	<0.0001
BMI	24.4 ± 5.9 (21–33)	26.8 ± 3.7 (21–33)	NS	NS	0.0008
WHR	0.87 ± 0.1 (0.73–1.06)	0.89 ± 0.1 (0.7–1.05)	NS	NS	NS
% Body Fat	23.3 ± 7.4 (7.9–42)	32.0 ± 7.7 (20.8–45)	0.0003	NS	NS
Fasting Glucose (mg/dl)	89 ± 9 (72–104)	94 ± 8 (79–117)	NS	0.0254	0.0005
Fasting Insulin (pmol/l)	50 ± 32 (23–167)	55 ± 25 (21–118)	NS	NS	0.0116
HOMA (μU/ml/mmol/L)	1.6 ± 1.2 (0.7–5.9)	1.9 ± 0.9 (0.6–4.0)	NS	NS	0.0028
OGTT 2h value (mg/dl)	96 ± 21 (59–139)	156 ± 16 (140–196)	<0.000001	NS	<0.0001
AUC_OGTT (mg/dl)	795 ± 144 (537–1,067)	1,066 ± 146 (831–406)	<0.000001	NS	<0.0001
Total Cholesterol (mmol/L)	4.92 ± 0.83 (3.5–7.1)	5.23 ± 0.78 (4.1–7.1)	NS	NS	NS
LDL (mmol/L)	3.0 ± 1.0 (1.9–5.0)	2.9 ± 1.4 (1.0–4.8)	NS	NS	NS
HDL (mmol/L)	1.5 ± 0.3 (0.8–2.2)	1.7 ± 0.4 (1.1–2.5)	0.04	NS	NS
Triglycerides (mmol/L)	0.9 ± 0.4 (0.4–2.1)	0.8 ± 0.2 (0.5–1.3)	NS	NS	0.0223
Triglyceride/HDL	0.7 ± 0.4 (0.3–1.7)	0.5 ± 0.3 (0.2–1.2)	NS	NS	0.0455

All participants were nondiabetic. Blood was drawn after an overnight fast. Data are expressed as means ± SD. Differences between groups were assessed by Student's *t* test. The final two shaded columns show paired t-tests for differences in the NGT group or in the IGT group from initial to final values.

AUC, Area Under the Curve; BMI, Body Mass Index; HDL, High Density Lipoproteins; IGT, Impaired Glucose Tolerance; LDL, Low Density Lipoproteins; n/a, Not applicable; NGT, Normal Glucose Tolerance; NS, Not Significant; OGTT, Oral Glucose Tolerance Test; WHR, Waist Hip Ratio.

### Sex as a biological variable

Forty-eight participants were recruited for this study, including 24 males and 24 females (Table 1). Baseline PAHSA and PAHOA isomer levels were compared between males and females. Changes over time and relationships with other metabolic parameters were studied in both sexes.

### Lipid extraction for PAHSA, PAHOA, and OAHSA measurements

Lipid extraction and analysis were performed as previously described (8). Briefly, sera samples were dounce homogenized in a mixture of 1.5 ml methanol, 1.5 ml chloroform, and 3 ml citric acid buffer. PAHSA standards were added to chloroform before extraction. The mixture was centrifuged, and the organic phase containing extracted lipids was dried under N2 before solid phase extraction. PAHSAs, PAHOAs, and Oleic Acid Hydroxy Stearic Acids (OAHSAs) were measured on an Agilent6410 Triple-Quad LC/MS. Extracted and fractionated samples were reconstituted in methanol; 10 ul was injected for analysis. Distinct PAHSA, PAHOA, and OAHSA species were resolved via isocratic flow at 0.15 ml/min for 60 min using 93:7 Methanol:Water with 5 mM ammonium acetate and 0.01% ammonium hydroxide as solvent (8). The separation, positional validation, and fragmentation pattern using these techniques have previously been published (8, 11, 28).

### Synthesis of (R, E)-10-(palmitoyloxy) octadec-8-enoic acid (10-PAHOA)

10-PAHOA was synthesized as follows. To a stirred solution of (R, E)-10-hydroxyoctanic-8-enoic acid (5 mg, 0.017 mmol, 1.0 equiv) in a mixture of dry methanol (2 ml) and benzene (2 ml) under the nitrogen atmosphere, trimethylsilyl diazomethane (0.2 ml, 0.4 mmol, 2M solution) was added. The reaction mixture was stirred for 20 min at 23°C, and after completion of the reaction, the solvents and reagents were distilled out under vacuum at 30° C to provide methyl (R, E)-10-hydroxyoctadec-8-enoate (1) (5 mg, 100%) in sufficient purity for the next step (without further purification) Rf = 0.3 (silica gel, 90:10 hexanes: EtOAc).

To a stirred solution of methyl (R, E)-10-hydroxyoctadec-8-enoate (1) (3 mg, 0.01 mmol; 1.0 equiv) in dry dichloromethane (3 ml), pyridine (5 μl, 0.06 mmol, 6 equiv) was added, and the reaction was cooled to 0 °C. Neat palmitoyl chloride (8 mg, 0.04 mmol, 4 equiv) was added after 5 min. The reaction mixture was stirred for 16 h at 23 °C. The solution was then concentrated, and the mixture was purified by column chromatography (7: 93 EtOAc: Hexanes) to yield the methyl (R, E)-10-(palmitoyloxy) octadec-8-enoate (2) (4 mg, 76%). Rf = 0.7 (silica gel, 90:10 hexanes: EtOAc); 1H NMR (600 MHz, CDCl3) δ 5.71–5.61 (m, 1H), 5.36 (dd, J = 15.4, 7.3 Hz, 1H), 5.18 (q, J = 6.9 Hz, 1H), 3.66 (s, 3H), 2.32–2.15 (m, 4H), 2.03–1.96 (m, 2H), 1.64–1.57 (m, 8H), 1.31–1.21 (m, 40H), 0.90–0.85 (m, 6H).

To a stirred solution of methyl (R, E)-10-(palmitoyloxy) octadec-8-enoate (2) (3 mg, 0.005 mmol, 1 equiv) in THF (2 ml), 1N lithium hydroxide (30 μl, 0.03 mmol, 6 equiv) solution was added, and the solution was stirred for 16 h. After completion of the reaction, the solution was diluted with EtOAc (10 ml). The organic layer was washed with pH-4 buffer solution (2 × 4 ml) and brine (5 ml). The organic layer was dried over sodium sulfate, filtered, and concentrated. The crude compound was purified by using column chromatography (12: 88 EtOAc: Hexanes) to yield the (R, E)-10-(palmitoyloxy) octadec-8-enoic acid (10-PAHOA) (1 mg, 34%). Rf = 0.4 (silica gel, 80:20 hexanes: EtOAc); 1H NMR (600 MHz, CDCl3) δ 5.70–5.62 (m, 1H), 5.36 (dd, J = 15.4, 7.4 Hz, 1H), 5.18 (q, J = 6.7 Hz, 1H), 2.35 (m, 2H), 2.28 (t, J = 7.5 Hz, 2H), 2.03–1.98 (m, 2H), 1.63–1.58 (m, 8H), 1.25 (s, 40H), 0.89–0.86 (m, 6H).

### GSIS studies

Human islets were cultured in human islet medium (Prodo Laboratories) containing 10% heat-inactivated serum supplemented with antibiotics. Human islets were serum-starved using KRB buffer for 3 h and treated with 9-PAHSA and 10-PAHOA (20 μM each) or DMSO (0.01%) for the last 1 h. Insulin was measured with an ELISA from Alpco Diagnostics (8).

### Glucose uptake studies in differentiated 3T3L1 adipocytes

3T3-L1 fibroblasts were cultured and differentiated as previously described (13). Insulin-stimulated glucose uptake studies in differentiated 3T3L1 adipocytes were initiated on Day 8 of differentiation after 24 h pretreatment with 9-PAHSA and 10-PAHOA (20 μM each) or vehicle (DMSO, 0.01%). 3T3L1 adipocytes were then serum-starved for 3 h, and after 20 min of incubation with the Krebs-Ringer-Hepes (KRH) buffer (13), adipocytes were stimulated with 5, 10, and 25 nM insulin for 20 min. Cells were later incubated with 1 μCi of [3H] deoxy-glucose for 10 min and washed with the KRH buffer supplemented with 200 mM glucose and 10 μM cytochalasin-B. Cells were then lysed with 0.5% SDS, and radioactivity of the lysed cells was measured. Protein concentration was measured by the Bicinchoninic Acid Protein Assay.

### LPS-stimulated cytokine secretion

Human peripheral blood mononuclear cells were purchased from the NIH repository. Cells were incubated with 9-PAHSA and 10-PAHOA (20 μM each) or DMSO (0.01%) for 15 min before LPS (10 ng/ml) stimulation. 24 h after LPS stimulation, media was collected for cytokine measurements. TNF-α, IL-12, and IL-6 levels were measured using human ELISA kits (BioLegend).

### Statistics

We summarized the sample characteristics by group (NGT to NGT and NGT to IGT) using mean and standard deviation for continuous variables and absolute and relative frequencies for categorical variables. We used independent samples *t* test to compare continuous variables and chi-square test to compare categorical variables between the groups; further on, we used paired samples *t* test to compare changes in clinical parameters from baseline to the final follow-up. We used Least Squares Multivariable Linear Regression to estimate the prediction of the final 2h-OGTT-glucose and change from baseline based on clinical parameters and PAHSA or PAHOA regioisomers and their changes. We performed nested model comparison and generated the receiver–operating characteristic (ROC) curves and the corresponding area under the curve (AUC) to determine whether PAHSAs or PAHOAs improve prediction of which participants will develop IGT versus those who will remain NGT. We did not include family history as a risk factor because all participants had first degree relatives with T2D. All reported *P*-values are two-tailed and are not adjusted for multiple testing, since the primary focus is the evaluation of prediction of 2h-OGTT levels based on the FAHFA concentrations. Statistical analyses were performed using GraphPad Prism-9.

### Study approval

The human study protocol was approved by the Ethical Committees at the Sahlgrenska Academy at the University of Gothenburg and was performed in accordance with the Declaration of Helsinki. The use of de-identified human samples was approved by the Institutional Review Board at Beth Israel Deaconess Medical Center.

## Results

Study participants were relatively young and had normal glucose tolerance at enrollment. Most had BMI and % body fat consistent with leanness in a North American or Scandinavian population (Table 1). 24 males and 24 females were enrolled. Over time, a greater percentage of females (18/24) developed IGT than males (4/24) (Table 1). The participants who developed IGT (NGT-IGT) differed from those who maintained NGT (NGT-NGT). NGT-IGT were on average 8 years older and had 8% greater body fat at baseline. Their mean 2h-OGTT glucose value was 24 mg/dl higher, albeit still in the normal range, and their average OGTT AUC was 12% higher than NGT-NGT (Table 1). All other clinical and metabolic characteristics were similar at baseline in the NGT and IGT groups (Table 1). At follow-up, the NGT-IGT group was slightly older, had 10% greater body fat, 50% higher 2h-OGTT value, 20% greater OGTT AUC, and 0.2 mM higher serum HDL compared to NGT-NGT (Table 2). Comparison of metabolic changes from baseline to follow-up showed the NGT-NGT group had a slightly higher fasting glucose at follow-up compared to enrollment (Table 2; fifth column). In NGT-IGT, BMI, WHR, fasting glucose, fasting insulin, HOMA, 2h-OGTT glucose, AUC during an OGTT, triglycerides, and triglyceride/HDL ratio (29) all increased (Table 2; last column).

**Fig. 1:**
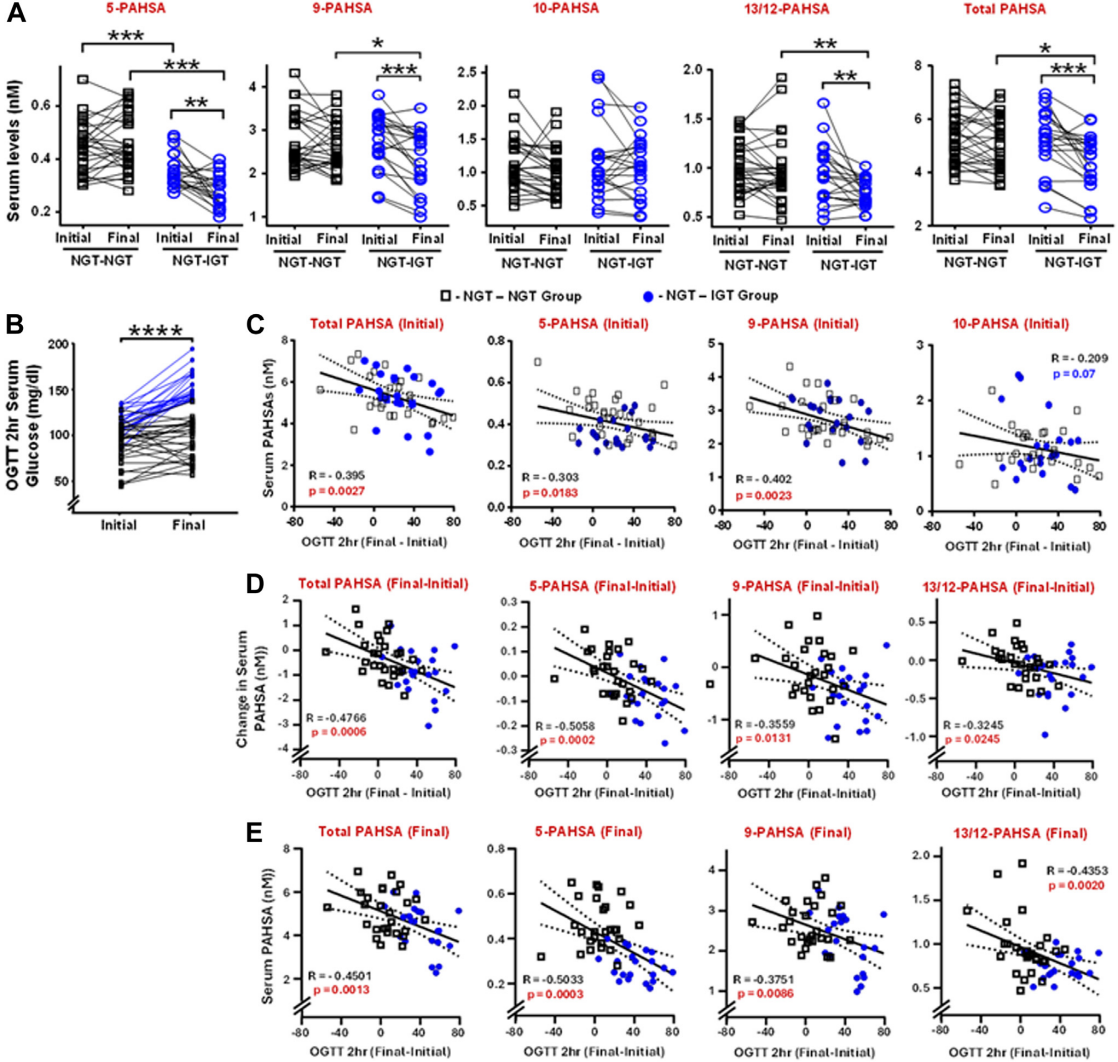
Serum PAHSA levels decrease over time in participants who start with normal glucose tolerance (NGT) and develop impaired glucose tolerance (IGT) (NGT-IGT) compared to participants who remain normal glucose tolerant throughout the study (NGT-NGT). Initial serum PAHSA levels, decreases in PAHSA levels and final PAHSA levels correlate with worsening glucose tolerance. A: Quantification of total PAHSAs and individual PAHSA regioisomers in NGT-NGT (black squares) and NGT-IGT (blue circles) male and female participants at baseline (initial) and follow-up (final). See Table 1 for metabolic characteristics. Data are means ± SEM. ∗*P* < 0.05; ∗∗*P* < 0.009; ∗∗∗*P* < 0.0001. Paired *t* test was performed within each group and unpaired *t* test was performed between groups. B: 2-h serum glucose values in all participants during an oral glucose tolerance test (OGTT) performed at baseline and follow-up. N = 48/group. Black lines are NGT-NGT participants. Blue lines are NGT-IGT participants. Data are means ± SEM. ∗∗∗∗*P* < 0.00005. Analysis was performed by paired *t* test. C: Correlation between initial (baseline) total PAHSAs and individual isomers with change in 2h-OGTT value (final – initial) in both male and female participants. D: Correlation between change in total PAHSAs and individual isomers (final – initial) with change in 2h-OGTT (final – initial) value in both male and female participants. E: Correlation between final total PAHSAs and individual isomers with change in 2h-OGTT (final – initial) value in both male and female participants. N = 21–27/group. For C–E, correlations were determined by Pearson linear regression analysis. P and R values are on individual graphs. Best fit linear correlation line is shown with 95% Confidence intervals (dotted lines).

Levels of total PAHSAs (sum of all regioisomers) and 9-, 10-, 12/13- regioisomers were similar between males and females at enrollment. However, females had slightly lower serum 5-PAHSA levels compared to males (Supplemental Fig. S1). We investigated whether PAHSA levels change over time and whether the levels predict worsening glucose tolerance. In NGT-NGT, follow-up levels of all PAHSA regioisomers and total PAHSAs were similar to initial levels (Fig. 1A). In contrast, for NGT-IGT, total PAHSAs and most PAHSA regioisomers except 10-PAHSA were lower at follow-up compared to initial levels (Fig. 1A). Thus, a reduction in serum PAHSA concentrations is associated with IGT development. Although 11-PAHSA has been reported to be present in human serum at low levels (22), with our methods, 11-PAHSA is at the limit of detection, and we cannot quantitate it reliably.

Participants who had higher initial 2h-OGTT values were more likely to develop IGT at follow-up compared to participants with lower initial 2h-OGTT values (Fig. 1B). Lower initial total PAHSAs and PAHSA regioisomers (5-, and 9-) were associated with worsening 2h-OGTT values (Fig. 1C). In addition, the magnitude of reduction in PAHSA levels from baseline to follow-up is also associated with worsening glucose tolerance (greater increase in 2h-OGTT) (Fig. 1D). Lower final concentrations of total and individual PAHSAs were also associated with worsening 2h-OGTT values (Fig. 1E). The correlations are highly significant for total PAHSAs and all regioisomers except 10-PAHSA with the strongest association with 5-PAHSA (Fig. 1D, E). Thus, initial PAHSA levels and change in PAHSA concentrations over time may predict worsening glucose tolerance and the development of IGT.

Lower initial serum total PAHSAs and a decrease in total PAHSAs predict worsening 2h-OGTT independent of sex, initial age, initial BMI, change in BMI (Table 3 and Supplemental Fig. S2), initial % body fat, change in % body fat (Supplemental Table S1 and Supplemental Fig. S2), initial fasting glucose, initial fasting insulin, and initial triglyceride/HDL ratio (Supplemental Table S2A–C). Initial 9-PAHSA and the change in 5-PAHSA and 9-PAHSA levels also predict the change in final 2h-OGTT glucose better than sex, initial age, initial BMI, change in BMI, initial % body fat, and change in % body fat (Table 3, Supplemental Table S1, and Supplemental Fig. S3). Change in 9- and 5-PAHSA also predicts worsening glucose tolerance independent of initial triglyceride/HDL ratio (Supplemental Table S3A, B). Since baseline 2h-OGTT value can predict worsening glucose tolerance, for each of the models described above, we adjusted for the initial 2h-OGTT and found similar predictive results to the non-adjusted analyses (not shown).

**Table 3:** Multivariable Linear Regression Analysis of initial and change in total PAHSAs, 5-PAHSA, 9-PAHSA, initial BMI, change in BMI with change in 2h-OGTT (final–initial) as outcome variable

	β (SE)	t	*P* Value	*R*^2^	Adjusted *R*^2^	F (*P* Value)
Independent Variables	Total PAHSAs			0.36	0.31	4.42 (0.0016)
Initial Total PAHSAs	−8.39 (3.78)	2.22	0.0321			
Change in Total PAHSAs	−15.11 (4.30)	3.51	0.0011			
Sex^a^: Male	−0.22 (7.31)	0.03	0.9765			
Age	0.77 (0.55)	1.39	0.1731			
Initial BMI	0.11 (1.17)	0.09	0.9279			
Change in BMI	2.76 (2.20)	1.25	0.2175			

	5-PAHSA			0.43	0.34	4.96 (0.0007)

Initial 5-PAHSA	−49.05 (37.30)	1.32	0.1960			
Change in 5-PAHSA	−142.8 (36.3)	3.94	0.0003			
Sex^a^: Male	3 (7.23)	0.18	0.8593			
Age	0.22 (0.53)	0.42	0.6776			
Initial BMI	0.41 (1.15)	0.36	0.7209			
Change in BMI	4.77 (2.20)	2.17	0.0359			

	9-PAHSA			0.32	0.22	3.15 (0.0127)

Initial 9-PAHSA	−13.27 (6.48)	2.05	0.0473			
Change in 9-PAHSA	−18.31 (7.66)	2.39	0.0216			
Sex^a^: Male	−1.52 (7.91)	0.19	0.8482			
Age	0.96 (0.56)	1.71	0.0959			
Initial BMI	0.26 (1.25)	0.21	0.8381			
Change in BMI	2.62 (2.30)	1.14	0.2625			

Analysis was performed in male and female participants combined. β Estimate, Standardized Coefficient Beta; F, F-statistic; SE, Standard Error; t, *t* test statistic.

a Reference category: “Female”.

We also tested whether adding PAHSAs to 2h-OGTT and other standard clinical parameters that are considered to be risk factors for IGT improved the prediction of worsening glucose tolerance. Compared to a model that includes only 2h-OGTT-glucose, initial BMI, change in BMI, sex and age, the inclusion of total PAHSAs at baseline and the change in total PAHSAs (or 5- or 9-PAHSAs) improves the prediction of final 2h-OGTT from adjusted *R*^2^ of 0.39 to 0.41, 0.54, and 0.43, respectively (Supplemental Table S4).

Following up on the analysis that showed that including PAHSAs improved the prediction of final 2h- OGTT, we performed ROC curves which demonstrated that the incorporation of PAHSAs enhances the ability to distinguish between participants who develop IGT versus those who remain NGT. Specifically, we tested whether PAHSAs improve the prediction of impaired glucose tolerance (IGT) better than the standard clinical biomarkers (initial BMI, change in BMI, initial HOMA, and change in HOMA), by performing nested model comparison and generating the ROC curves and the corresponding AUC. Compared to the model that includes only standard clinical biomarkers, the inclusion of initial or change in total PAHSAs, 5-PAHSA, or 9-PAHSAs improves the prediction of IGT, from AUC of 0.73 to 0.83, 0.99, and 0.83, respectively (Supplemental Fig. S4).

We also analyzed the impact of follow-up duration on the predictive value of worsening 2h-OGTT. Initial total PAHSAs and the change in total PAHSAs predict the change in 2h-OGTT glucose independent of follow-up duration (Supplemental Table S5A). The change in total PAHSAs also predicts the final 2h-OGTT glucose value independent of follow-up duration (Supplemental Table S5B). Thus, the length of follow-up is not a confounding factor in this study. Many other clinical parameters do not correlate with IGT development in this relatively young, lean cohort. Change in PAHSA levels correlates better with worsening OGTT than the triglyceride/HDL ratio and other parameters (Supplemental Fig. S5). Also, initial total PAHSA, initial 5-PAHSA, change in total PAHSA, change in 5-PAHSA, and final 5-PAHSA levels correlate more strongly with worsening OGTT glucose than % body fat (Supplemental Fig. S6 and Supplemental Table S6). Overall, initial and/or change in 5-PAHSA, 9-PAHSA, and Total PAHSAs are associated with, and predict, worsening glucose tolerance over time better than other clinical parameters.

**Fig. 2:**
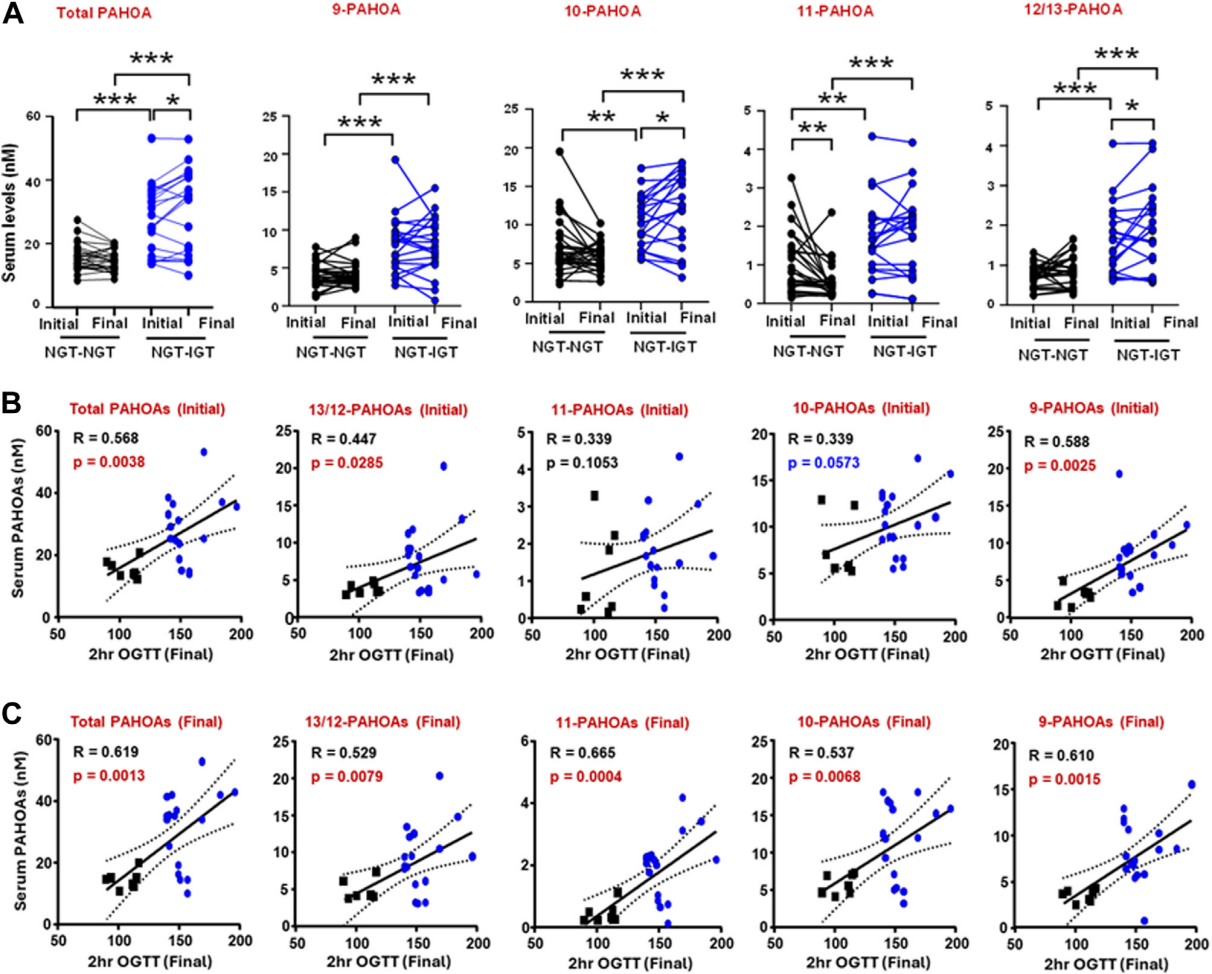
Serum total PAHOA and most PAHOA regioisomer levels are increased at baseline (initial) and follow-up (final) in participants who develop impaired glucose tolerance (NGT-IGT) compared to participants who remain normal glucose tolerant (NGT-NGT). Both initial and final PAHOA levels correlate with worsening glucose tolerance. A: Quantification of serum total PAHOAs and individual PAHOA regioisomers in all male and female participants at baseline (Initial) and follow-up (Final). See Table 1 for metabolic characteristics. N = 21–27/group. Data are means ± SEM. ∗*P* < 0.05; ∗∗*P* < 0.009; ∗∗∗*P* < 0.0001. A paired *t* test was performed within each group, and unpaired *t* test was performed between groups. B: Correlation between initial total PAHOA and individual regioisomers with follow-up 2h-OGTT value and in female participants. N = 24. C: Correlation between final total PAHOA and individual regioisomers with follow-up 2h- OGTT value and in female participants. N = 24. For B, C, correlations were determined by Pearson linear regression analysis. P and R values are on individual graphs. Best-fit linear correlation line is shown with 95% Confidence intervals (dotted lines).

Since PAHOAs are one of the most abundant FAHFA families in human serum, we also investigated whether serum PAHOA levels predict worsening glucose tolerance. Since PAHOAs may exist with either a single or double bond, we use the term “PAHOAs” to indicate that we are measuring endogenous PAHOAs with either a single or double bond. Initial and final serum total PAHOAs and 9-, 10-, 11-, 12/13- regioisomers were lower in males compared to females (Supplemental Fig. S7). In participants who remained NGT, total-PAHOAs and most PAHOA isomer levels, except 11-PAHOA, did not change between enrollment and follow-up. 11-PAHOA levels in NGT-NGT participants were lower at the follow-up compared to baseline (Fig. 2A). For the participants who developed IGT, the initial and follow-up total PAHOAs and all regioisomer levels were higher compared to NGT-NGT participants (Fig. 2A). In addition, in the NGT-IGT group the total PAHOAs, and 10- and 12/13-PAHOA levels but not 9-, and 11- regioisomers were higher at follow-up compared to baseline (Fig. 2A). This indicates that higher serum levels of total PAHOAs and regioisomers are associated with IGT development in the whole group. In female participants, higher initial levels of total PAHOAs and 12/13- and 9-PAHOA are associated with worsening 2h-OGTT values at follow-up, with a strong tendency for 10-PAHOA (Fig. 2B). Higher follow-up total PAHOAs and all PAHOA regioisomers in female participants are also associated with worsening 2h-OGTT, with the strongest association with 11-PAHOA (Fig. 2C).

Only four male participants developed IGT compared to 17 females. Hence, when analyzing the males alone, the initial total PAHOA and regioisomer levels did not show a statistically significant relationship with 2h-OGTT at follow-up (Supplemental Fig. S8A), but the final total PAHOA and 10-PAHOA levels correlated with worse glucose tolerance (Supplemental Fig. S8B). When we combine male and female data, the initial and final total PAHOAs and all regioisomers strongly correlate with final 2h-OGTT (Supplemental Table S6). The change in total PAHOA and 11- and 10-PAHOA levels in male and female participants combined also correlated strongly with follow-up 2h-OGTT (Supplemental Table S7). In summary, the greater the serum PAHOA levels at baseline and the end of the study, the more glucose-intolerant the participant becomes over the study period.

Similar to PAHSA analysis, we constructed a linear correlation matrix for the clinical characteristics and serum PAHOA levels to control for potential clinical confounders of increased 2h-OGTT (Supplemental Fig. S9). Based on the multivariable regression, higher initial total serum PAHOA levels predict higher final 2h-OGTT, independent of initial BMI, change in BMI, initial % body fat, or change in % body fat (Table 4 and Supplemental Table S8). This effect is also independent of initial fasting glucose, initial fasting insulin, and initial triglyceride/HDL ratio (Supplemental Table S9A–C). We also tested whether PAHOAs improve the prediction of which participants will develop IGT better than the standard clinical biomarkers by performing a nested model comparison. Compared to the model that includes only standard clinical biomarkers (initial BMI, change in BMI, initial HOMA, and change in HOMA), the inclusion of initial total PAHOAs improves the prediction of which participants will develop IGT, from AUC of 0.73 to 0.91 (*P* = 0.0248) (Supplemental Fig. S10).

**Table 4:** Multivariable Linear Regression Analysis of initial total PAHOAs, initial BMI, and change in BMI with final 2h-OGTT as outcome variable

	β (SE)	t	*P* Value	*R*^2^	Adjusted *R*^2^	F (*P* Value)
	Final 2h–OGTT Value					
Independent Variables				0.49	0.43	4.42 (<0.0001)
Initial total PAHOAs	0.99 (0.49)	2.01	0.0503			
Initial BMI	−0.53 (1.40)	−0.39	0.7009			
Change in BMI	0.21 (2.61)	0.08	0.9335			
Age	1.56 (0.67)	2.32	0.0249			
Sex^a^: Male	−24.34 (8.73)	2.01	0.0080			

Analysis was performed in male and female participants combined. β Estimate, Standardized Coefficient Beta; F, F-statistic; SE, Standard Error; t, *t* test statistic.

a Reference category: “Female”.

When adjusting for initial 2h-OGTT, unlike with PAHSAs, the effect of PAHOAs on final 2h-OGTT was no longer significant, which can be potentially attributed to the strong correlation between initial total PAHOAs and initial 2h-OGTT (R = 0.48; *P* = 0.0007). This is stronger than the correlation between initial 2h-OGTT and total PAHSAs (R = 0.11; *P* = 0.4330); 5-PAHSAs (R = −0.30; *P* = 0.0413); and 9-PAHSAs (R = 0.21; *P* = 0.1422). Overall, in this population of high-risk subjects, higher levels of serum total PAHOAs and PAHOA regioisomers are associated with, and predict, worsening glucose tolerance.

**Fig. 3:**
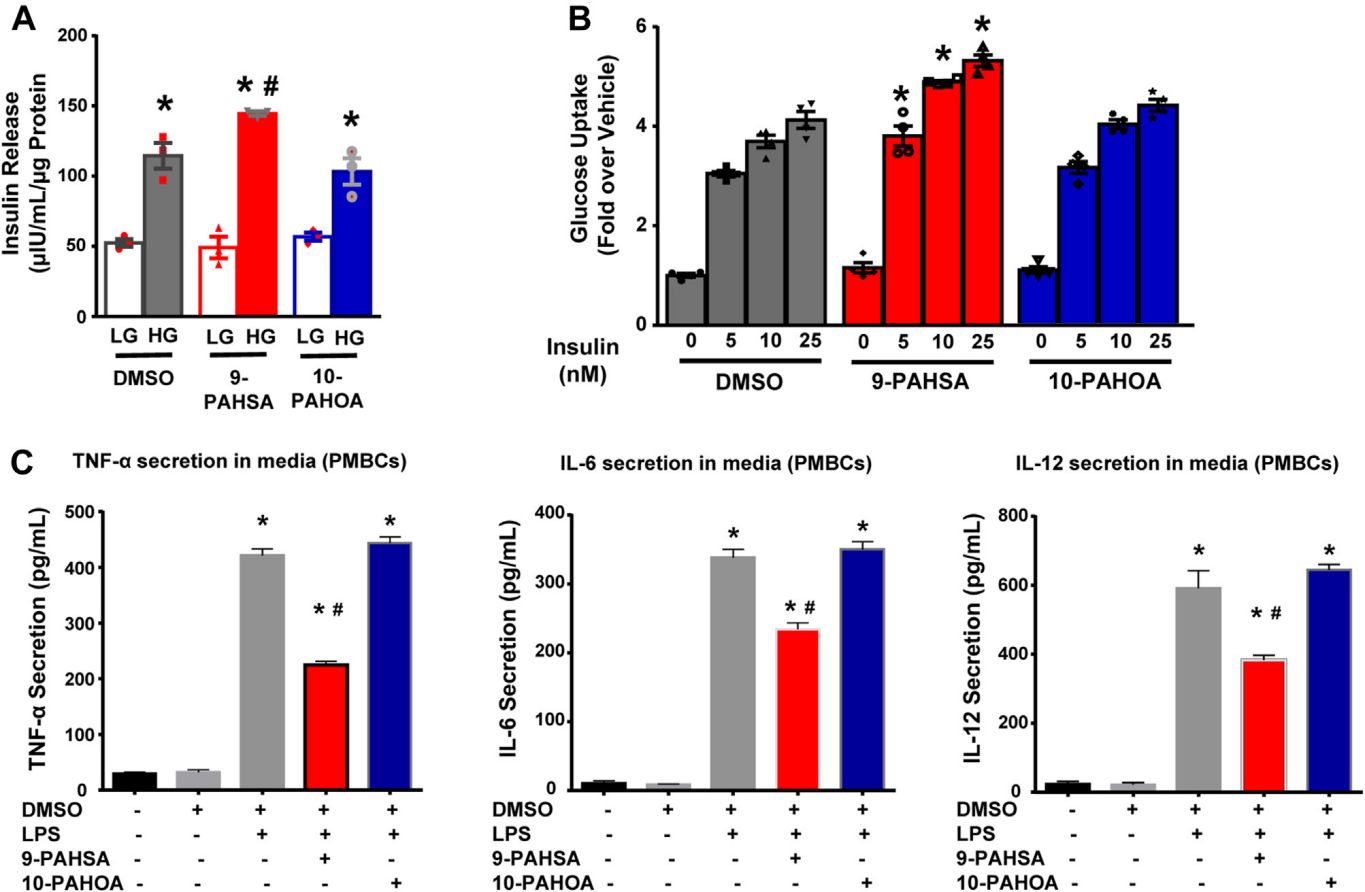
A: Glucose-stimulated insulin secretion from human islets. Insulin secretion was performed in primary human islets from two different non-diabetic donors. The islets were preincubated with 9-PAHSA (20 uM), 10-PAHOA (20 μM) or DMSO for 1 h in KRB and then exposed to low glucose (2.5 mM; LG) or high glucose (20 Mm; HG) concentrations in the continued presence of 9-PAHSA (20 μM), 10-PAHOA (20 μM) or DMSO for 45 min N = 50 islets/well and three wells per condition. Data are the means ± SEM. ∗*P* < 0.05 vs. respective low glucose; #*P* < 0.05 vs. high glucose DMSO by one-way ANOVA. B: Insulin-stimulated glucose uptake. Differentiated 3T3L1 adipocytes were treated with 9-PAHSA (20 μM), 10-PAHOA (20 μM), or DMSO for 24 h and then glucose transport was measured. N = 4–6 wells/condition. ∗*P* < 0.05 vs. DMSO. C: Effects of 9-PAHSA and 10-PAHOA on LPS-induced cytokine secretion from human peripheral blood mononuclear cells (PBMCs). PBMCs were preincubated with 9-PAHSA (20 μM), 10-PAHOA (20 μM) or DMSO for 15 min before LPS (10 ng/ml) stimulation. Cells were exposed to LPS for 24 h in continuous presence of 9-PAHSA, 10-PAHOA or DMSO. At the end of the treatment, cell culture media was collected for measurement of TNF-α, IL-6 and IL-12 secretion. N = 3–4 wells/condition. Data are means ± SEM and are representative of two independent studies. ∗*P* < 0.05 versus DMSO group; #*P* < 0.05 versus LPS-induced DMSO group by one-way ANOVA with Tukey's multiple comparison test. LPS, lipopolysaccharide; PAHSA, palmitic acid hydroxy stearic acid; PAHOA, Palmitic acid hydroxy oleic acid.

Since lower serum PAHSA levels but high PAHOA levels predict worsening glucose tolerance, we hypothesized that PAHOAs may not have the same beneficial metabolic effects that PAHSAs have. We tested the effects of 10-PAHOA, which had the highest correlation with 2h-OGTT value (Supplemental Table S7), on three key biologic processes that contribute to the antidiabetic effects of PAHSAs (8, 13). In isolated human islets, neither 9-PAHSA nor 10-PAHOA potentiated glucose-stimulated insulin secretion (GSIS) at low glucose (Fig. 3A). However, 9-PAHSA but not 10-PAHOA, potentiated insulin secretion in the presence of high glucose (Fig. 3A). Furthermore, neither 9-PAHSA nor 10-PAHOA stimulated glucose transport in the absence of insulin (basal) in adipocytes (Fig. 3B). However, 9-PAHSA, but not 10-PAHOA, potentiated glucose transport at submaximal and maximal insulin concentrations (Fig. 3B). In human peripheral blood mononuclear cells, LPS treatment increased TNF-α, IL-6, and IL-12 secretion and 9-PAHSA significantly attenuated this, while 10-PAHOA had no effect (Fig. 3C). These data demonstrate that 10-PAHOA does not have the beneficial anti-diabetic and anti-inflammatory effects that 9-PAHSA has. Since Oleic Acid Hydroxystearic Acids (OAHSAs) are also relatively abundant in human serum, we measured levels for total, 12-, 10-, and 9-OAHSA regioisomers in these participants. There were no differences in the initial OAHSA concentrations or the change over time in NGT-IGT compared to NGT-NGT.

Overall, these data show that low baseline serum PAHSA levels or a decrease over time predicts worsening glucose tolerance independent of age, sex, BMI, % body fat, change in BMI, change in % body fat, fasting glucose, fasting insulin, or triglyceride/HDL ratio. In addition, higher serum PAHOA concentrations at baseline predict worsening glucose tolerance independent of BMI, % body fat, change in BMI, change in % body fat, fasting glucose, fasting insulin, or triglyceride/HDL ratio. There is some specificity since OAHSAs do not predict glucose tolerance (data not shown), and some PAHSA and PAHOA regioisomers have stronger predictive value than others. Thus, concentrations of total and most PAHSA and PAHOA regioisomers could be used as biomarkers for the development of IGT and would be useful even in lean, relatively young people.

## Discussion

FAHFAs are a novel class of endogenous bioactive lipids; some sub-families, such as PAHSAs have anti-diabetic and anti-inflammatory effects (8). PAHSA levels correlate highly with insulin sensitivity in humans (8), but whether PAHSA levels can predict worsening glucose tolerance and/or risk of T2D is unknown. There is also no information about whether PAHOA or OAHSA regioisomer levels are altered in insulin-resistant humans or predict the development of IGT or T2D.

We found that baseline and final serum total PAHSA levels and most regioisomers, and a decrease in PAHSA levels over time, are strongly associated with worsening glucose tolerance in first-degree relatives of individuals with T2D. This relationship is preserved after multivariable analysis, controlling for age, sex, and other baseline clinical characteristics that are often associated with IGT and T2D risk, such as BMI, % body fat, fasting glucose, fasting insulin, and triglyceride/HDL ratio. In contrast, the concentrations of initial and final total PAHOAs and all PAHOA regioisomers were higher in participants who developed IGT than in those who remained NGT. The higher initial levels of total PAHOA and PAHOA regioisomers are associated with worsening 2h-OGTT independent of BMI, % body fat, change in BMI, or change in % body fat. It is not obvious why low PAHSAs but high PAHOAs predict IGT, except that most PAHSAs have beneficial metabolic effects (13) which we do not see with the PAHOA regioisomer that we tested in three biological activity assays in this study. Specificity among FAHFA families is further demonstrated by the fact that we found no difference in OAHSA concentrations between NGT-NGT and NGT-IGT participants.

Thus, PAHSA and PAHOA concentrations initially and over time (ie 3–12 years) could be used as biomarkers for worsening glucose tolerance and increased risk for IGT and T2D. This would be especially useful in leaner people in whom other traditional biomarkers are less predictive. Impaired glucose tolerance is a major risk factor for cardiovascular disease as well as for diabetes. Hence, our findings have important implications also for cardiovascular disease. These data raise the intriguing mechanistic possibility that low PAHSA levels and/or high PAHOA levels may contribute to diabetes development. There are multiple physiological mechanisms by which higher PAHSA concentrations could play a role in maintaining normal glucose tolerance, including insulin-sensitizing effects (8, 11, 12), augmentation of glucose-stimulated insulin secretion (8, 11, 13, 16), and anti-inflammatory effects (8, 13, 16). An intervention trial is warranted to determine whether increasing PAHSA levels could prevent IGT and T2D.

One limitation of this study is the small BMI changes and the small range in BMI in this middle-aged population. However, this is an advantage because our study is naturally controlled for BMI changes, and it offers a biomarker for people who are not obese. Another limitation is the limited genetic diversity of this Swedish study population relative to the diversity in many other countries. It will be interesting to determine whether serum PAHSA and PAHOA levels are good biomarkers for worsening glucose tolerance in other genetically and metabolically diverse groups.

In conclusion, lower serum PAHSA levels or a fall over time predict worsening glucose tolerance independent of age, sex, BMI, % body fat, change in BMI, change in % body fat, fasting glucose, fasting insulin, or triglyceride/HDL ratio, whereas initial elevated PAHOA levels predict worsening glucose tolerance. Whether these levels contribute to diabetes pathogenesis is unknown. However, since PAHSAs have beneficial effects on insulin sensitivity and insulin secretion, a fall in PAHSA levels could contribute to T2D pathogenesis, and maintaining PAHSA levels might be beneficial to prevent or ameliorate T2D. Importantly, PAHSA and PAHOA levels appear to be useful biomarkers for IGT development.

## Data availability

All study data are included in the article and supplementary materials.

## Supplemental data

This article contains supplemental data.

## Conflict of interest

The authors declare the following financial interests/personal relationships which may be considered as potential competing interests: I. S. and B. B. K. are inventors on the following patents: Lipids that Increase Insulin Sensitivity and Methods of Using the Same” (patent no. 20180194714), and Fatty Acid Esters of Hydroxy Fatty Acids (FAHFAs) for Use in the Treatment of Type 1 Diabetes (patent no. 20190151276).
