# Supplementary material for: Specific FAHFAs predict worsening glucose tolerance in non-diabetic relatives of people with Type 2 diabetes
Source: J Lipid Res. 2025 May 5;66(7):100819. doi: 10.1016/j.jlr.2025.100819 (PMC12271064; doi:10.1016/j.jlr.2025.100819)
Supplement: Supplemental data [file mmc1.pdf]

Supplementary Appendix to:

Specific FAHFAs Predict Worsening Glucose Tolerance in Non-Diabetic Relatives of People with Type 2 Diabetes

Ismail Syed[1], Ken Sluis[1a], Pratik Aryal[1b], Zachary Solomon[1c], Rucha Patel[1], Srihari Konduri[3], Dionicio Siegel[3], Ulf Smith[2], and Barbara B Kahn[1*].

1. Department of Medicine, Beth Israel Deaconess Medical Center and Harvard Medical School, Boston, Massachusetts, USA.
2. The Lundberg Laboratory for Diabetes Research, Departments of Molecular and Clinical Medicine, Institute of Medicine, Sahlgrenska Academy at the University of Gothenburg, Gothenburg, Sweden.
3. Skaggs School of Pharmacy and Pharmaceutical Sciences, University of California San Diego, La Jolla, CA, 92093, USA.

*To whom correspondence should be addressed at: bkahn@bidmc.harvard.edu

Barbara B. Kahn, MD

[a]Current address: Central Maine Endocrinology and Diabetes Center, Lewiston, ME 04240.

[b]Current address: Cellarity, Somerville, MA 02143.

[c]Current address: RUSH Copley Medical Group Dermatology, Aurora, IL, 60504.

Contents

Supplementary Figures:

Figure S1: Total PAHSAs and most of the PAHSA regioisomers are similar at baseline (Initial) in male and female participants…………………………………………………………………….…...**4**

Figure S2: Heat map showing all linear correlations between measured PAHSA levels and other parameters………………………….……………………………………………………...**5**

Figure S3: Linear correlations between 5-PAHSA and selected derivative parameters…………**6**

Figure S4: Receiver Operating Characteristic curves and Area Under the Curve for initial and change in total PAHSAs or 5-PAHSA or 9-PAHSA and selected derivative parameters……..….. **7**

Figure S5: Linear correlations between change in total PAHSAs and individual PAHSA isomer levels and selected clinical parameters…………………………………………………………...**8**

Figure S6: Multivariable collinearity analysis between selected parameters and initial serum total PAHSA levels or final 5-PAHSA levels………………………..……………………..…………….**9**

Figure S7: Total PAHOAs and all PAHOA regioisomer levels are higher in females compared to males at baseline (Initial) and follow-up (Final)……………………………………………….…….**10**

Figure S8: Final total PAHOA and 10-PAHOA regioisomer levels but not others correlate with worsening glucose tolerance in male participants…………………………………………….…….**11**

Figure S9: Linear correlations between serum PAHOA levels and selected parameters………**12**

Figure S10: Receiver Operating Characteristic curves and Area Under the Curve (AUC) for initial total PAHOAs and selected derivative parameters…………………………..……………**13**

Supplementary Tables:

Table S1: Multivariable Linear Regression Analysis of initial and change in total PAHSAs, 5-PAHSA, 9-PAHSA, initial % body fat, change in % body fat, Age and Sex with change in 2hr-OGTT (final-initial) as outcome variable…………………………….……………………………..**14**

Table S2: Multivariable Linear Regression Analysis of initial and change in total PAHSAs, initial fasting insulin, initial fasting glucose, and initial triglycerides/HDL with change in 2hr-OGTT (final-initial) as outcome variable………………………………………………………………….…**15**

Table S3: Multivariable Linear Regression Analysis of initial and change in 5-PAHSA and 9-PAHSA and initial triglyceride/HDL with change in 2hr-OGTT (final-initial) as outcome variable……………..………………………………………………………………………………**16**

Table S4: Multivariable Linear Regression Analysis of initial and change in total PAHSAs, 5-PAHSA, 9-PAHSA, initial BMI, change in BMI, Age and Sex with change in final 2hr-OGTT as outcome variable……………….………………………………………………………………..……**17**

Table S5: Multivariable Linear Analysis of initial total PAHSAs, change in total PAHSAs and follow-up time with change in 2hr-OGTT value (Final – Initial) or Final 2hr-OGTT value being the dependent variable……………………...……………………………………….……………**18**

Table S6: Pearson correlations for serum PAHSA levels with Final 2hr-OGTT ……….……..…**19**

Table S7: Pearson correlations for serum PAHOA levels with Final 2hr-OGTT……..……..……**20**

Table S8: Multivariable Linear Regression Analysis of initial total PAHOAs, initial % body fat, change in % body fat, Age and Sex with final 2hr-OGTT value as outcome variable……….……..**21**

Table S9: Multivariable Linear Analysis in all participants of initial serum total PAHOA levels, initial fasting insulin, initial fasting glucose, and initial triglyceride/HDL with final 2hr-OGTT value as outcome variable……………………………………………………………….…………………..**22**

<u>Supplementary Figures and Tables</u>:

<u>Supplementary Figure 1</u>: Total PAHSAs and PAHSA regioisomers except 5-PAHSA are similar at baseline (Initial) in male and female participants.

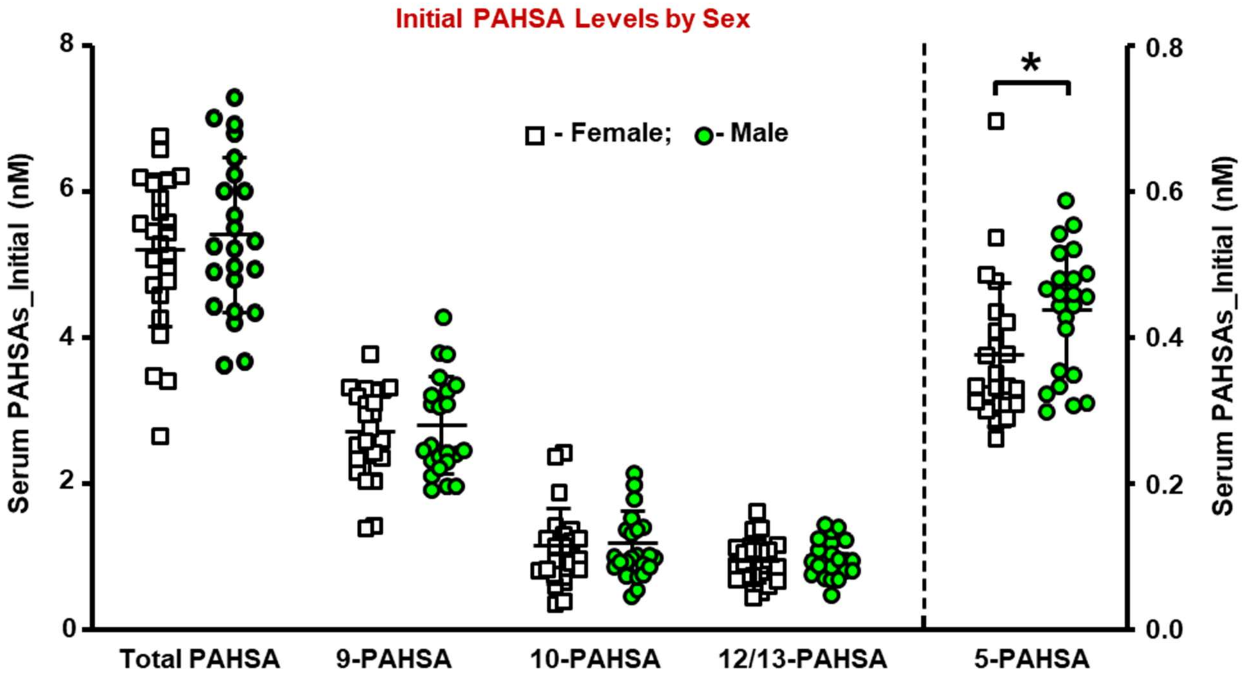

Quantification of total PAHSAs and individual PAHSA regioisomers in serum of male and female participants at baseline (see Table 1A for metabolic characteristics). n = 24/group. Data are means ± SEM. * $p<0.05$ for 5-PAHSA levels in females vs. males. Analysis was performed by Two-tailed t-test.

Supplementary Figure 2: Heat map showing all linear correlations between measured PAHSA levels and other parameters.

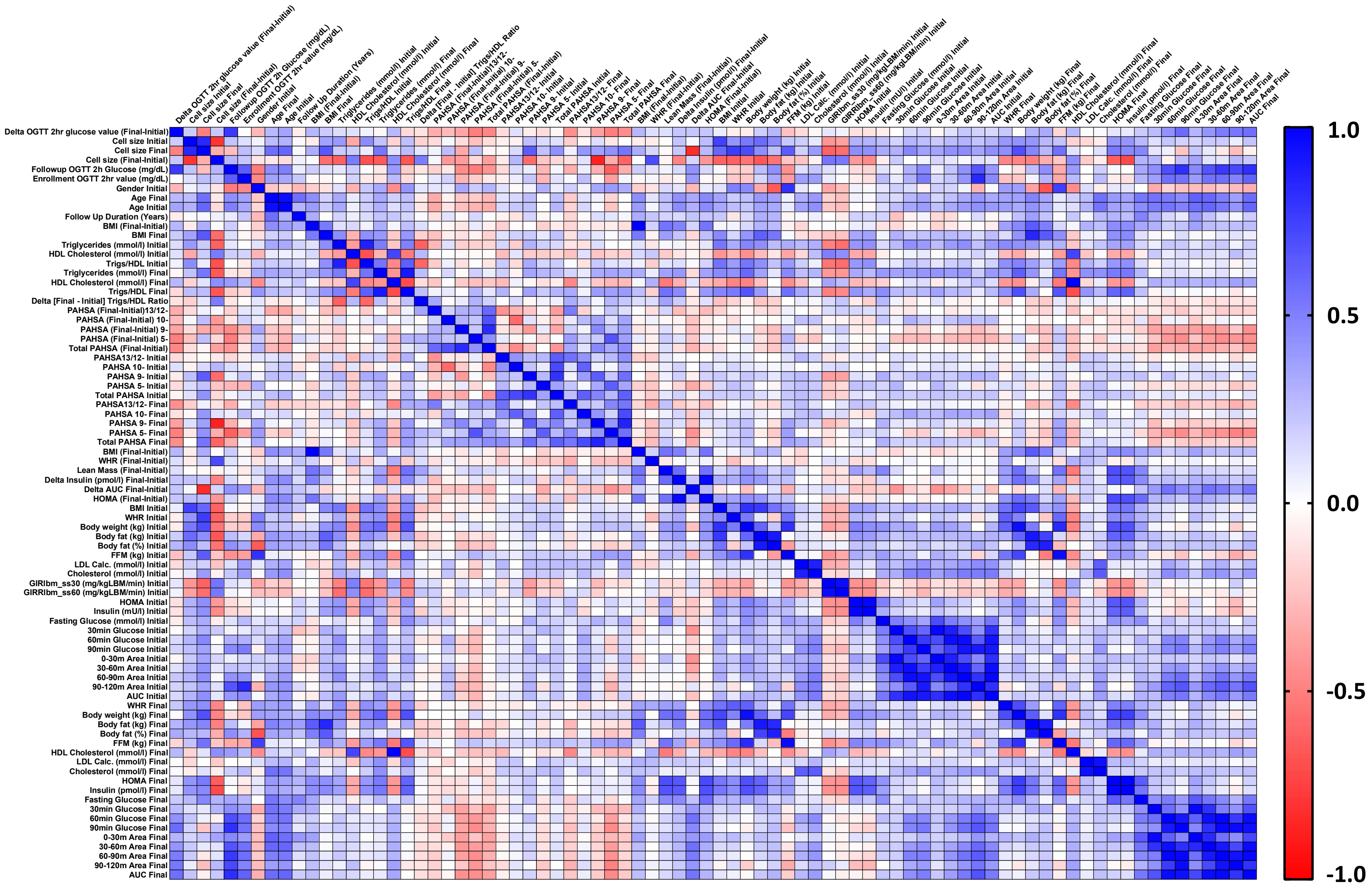

A linear correlation analysis was performed on all the clinical characteristics and serum total PAHSAs and individual regioisomers. The correlation intensity is indicated with red and blue. The darker the blue color, the more positively the parameters correlate with impaired glucose tolerance. The darker the red color, the more negatively the parameters correlate with impaired glucose tolerance.

Supplementary Figure 3: Linear correlations between 5-PAHSA and selected derivative parameters.

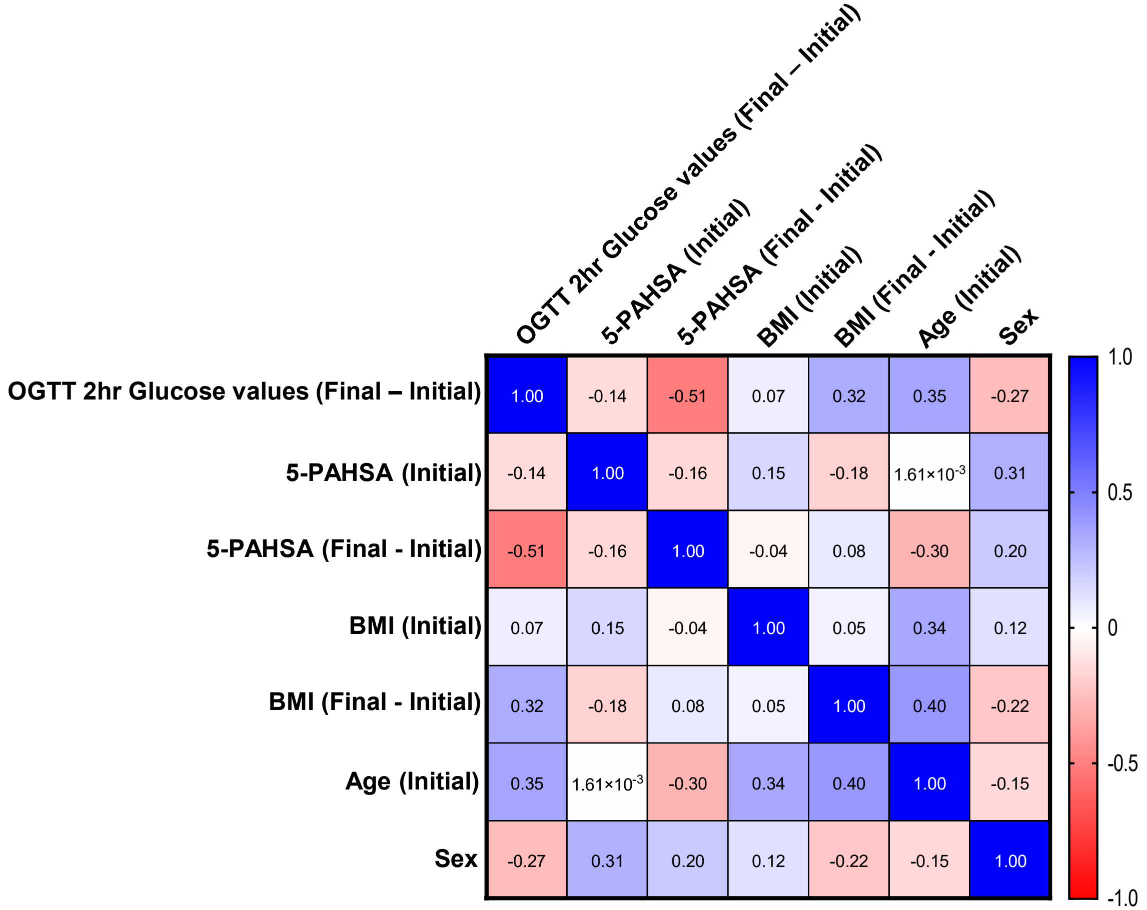

A linear correlation analysis was performed on selected clinical characteristics and 5-PAHSA levels. The “R” values for each correlation are in the boxes. The correlation intensity is indicated in red and blue. The darker the blue color, the more positively the parameters correlate with impaired glucose tolerance. The darker the red color, the more negatively the parameters correlate with impaired glucose tolerance.

<u>Supplementary Figure 4</u>: Receiver Operating Characteristic curves and Area Under the Curve (AUC) for initial and change in total PAHSAs or 5-PAHSA or 9-PAHSA and selected derivative parameters.

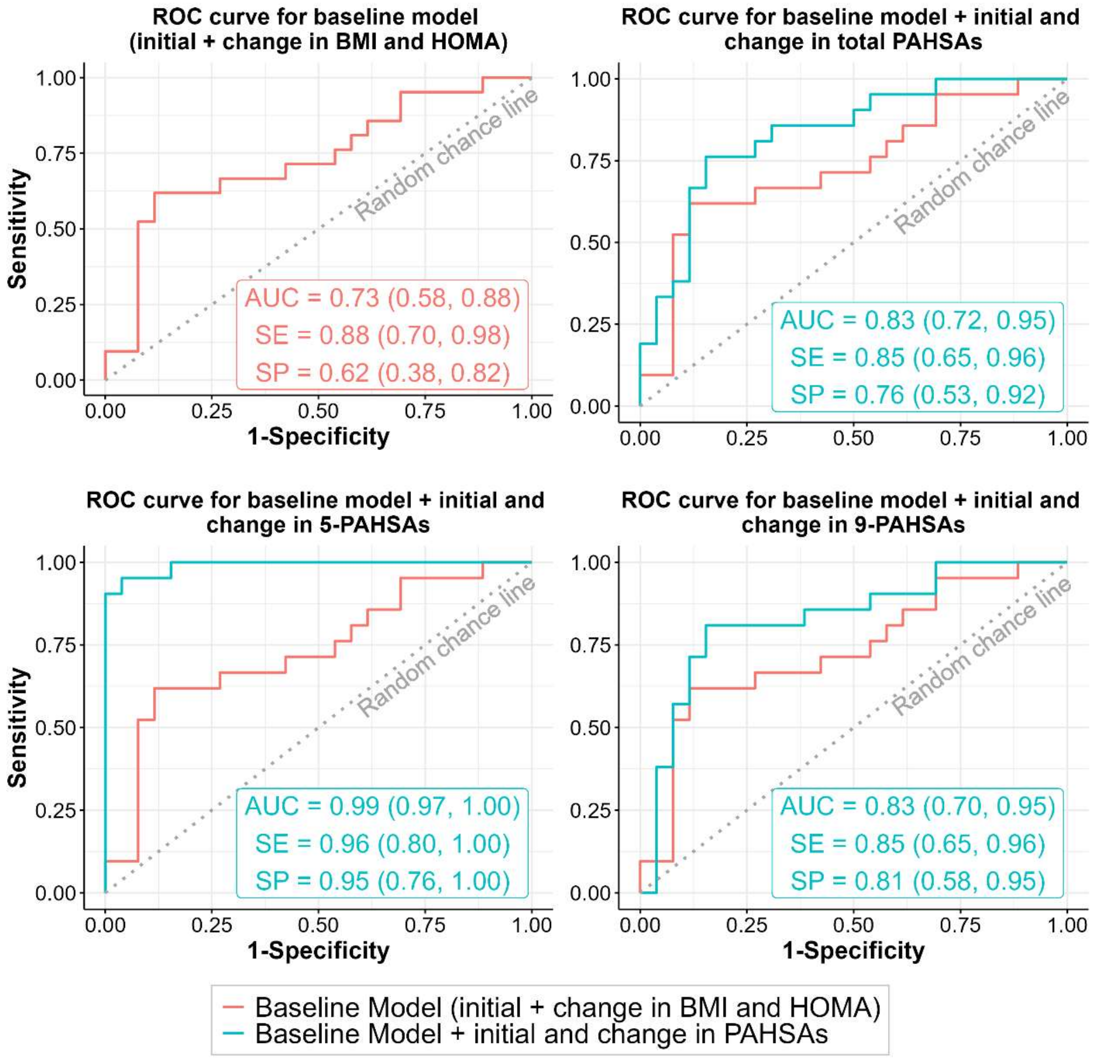

The measures are reported as estimated value (95% Confidence Interval). ROC: Receiver Operating Characteristic; AUC: Area under the Curve; SE: Sensitivity; SP: Specificity; 1-Specificity: False Positive Rate

Supplementary Figure 5: Linear correlations between change in total PAHSAs and individual PAHSA isomer levels and selected clinical parameters.

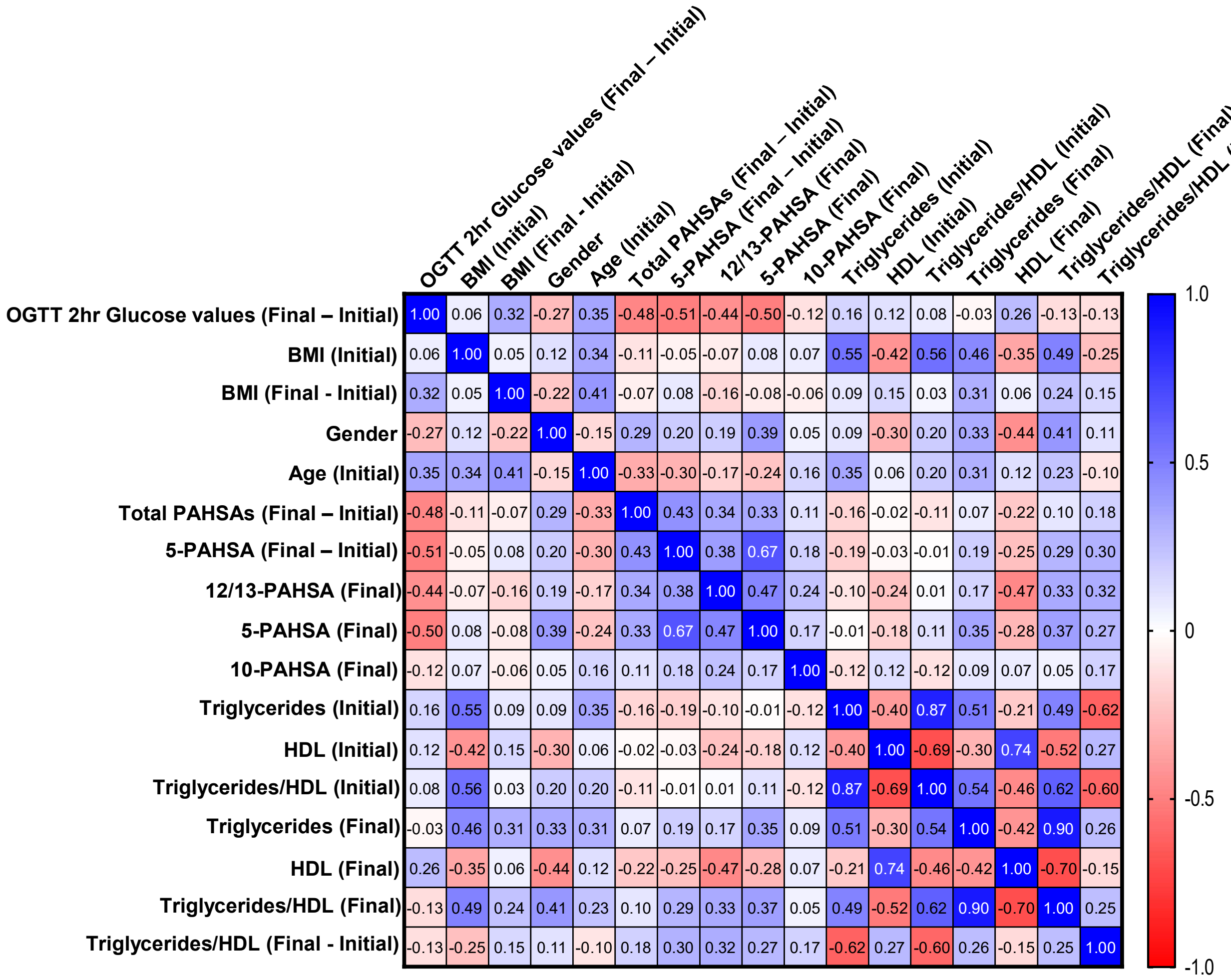

Pearson linear correlation analysis was performed on the selected clinical characteristics, and serum total PAHSAs or individual PAHSA regioisomers. The "R" values for each correlation are in the boxes. Collinear Pairs, R > 0.80. The correlation intensity is colored in red and blue. The darker the blue color, the more positively the parameters correlate with impaired glucose tolerance. The darker the red color, the more negatively the parameters correlate with impaired glucose tolerance.

<u>Supplementary Figure 6</u>: Multivariable collinearity analysis between selected parameters and initial serum total PAHSA levels or final 5-PAHSA levels.

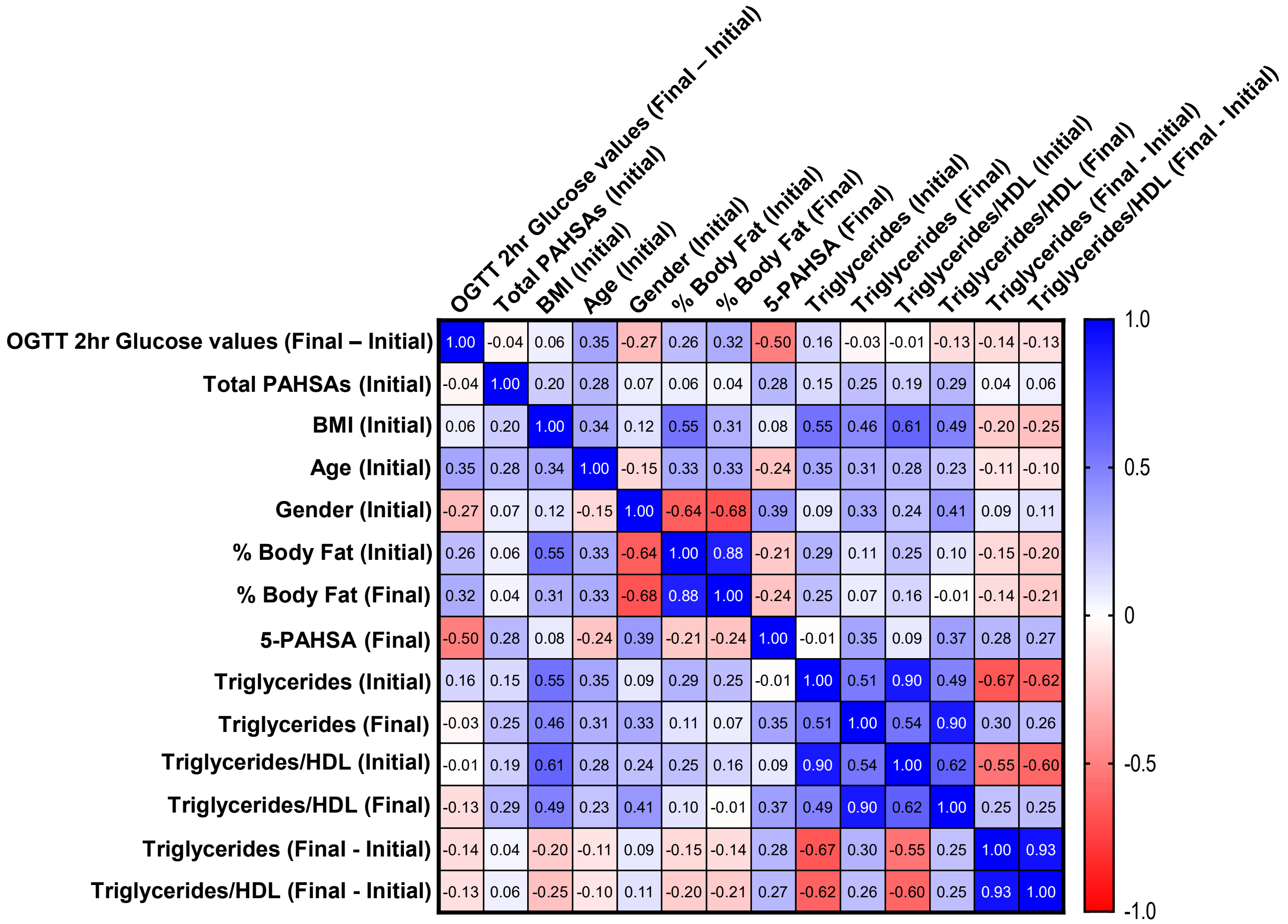

The “R” values for each correlation are in the boxes. Collinear Pairs, R > 0.80. The correlation intensity is colored in red and blue. The darker the blue color, the more positively the parameters correlate with impaired glucose tolerance. The darker the red color, the more negatively the parameters correlate with impaired glucose tolerance.

<u>Supplementary Figure 7</u>: Total PAHOAs and all PAHOA regioisomer levels are higher in females compared to males at baseline (Initial) and follow-up (Final).

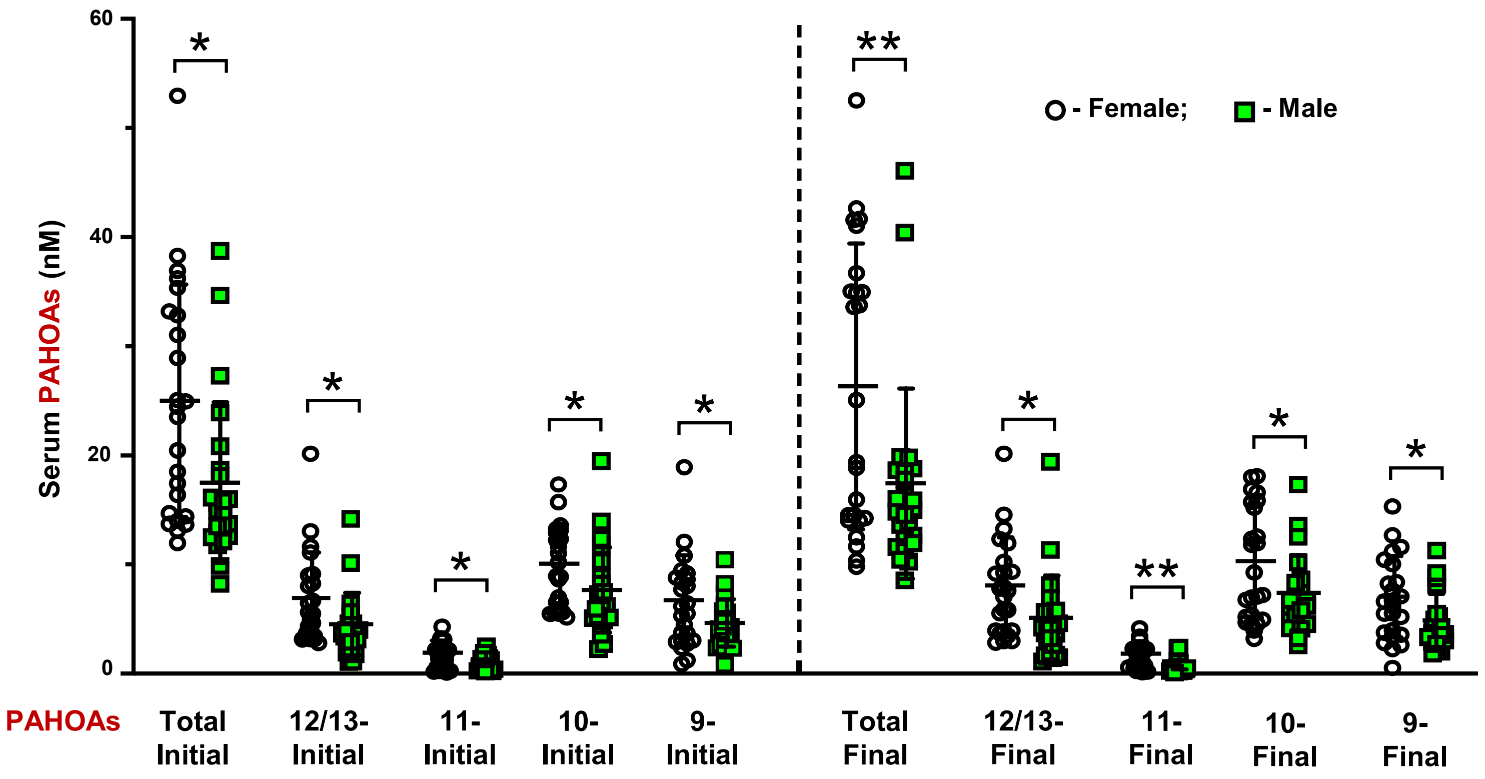

Quantification of total PAHOAs and individual PAHOA regioisomers in serum of male and female participants at baseline (Initial) and follow-up (Final). See Table 1A for metabolic characteristics. All participants were NGT at baseline. n = 24/group. Data are means ± SEM. * $p<0.05$; ** $p<0.009$. Analysis was performed by Two-tailed t-test.

Supplementary Figure 8: Final total PAHOA and 10-PAHOA regioisomer levels but not others correlate with worsening glucose tolerance in male participants.

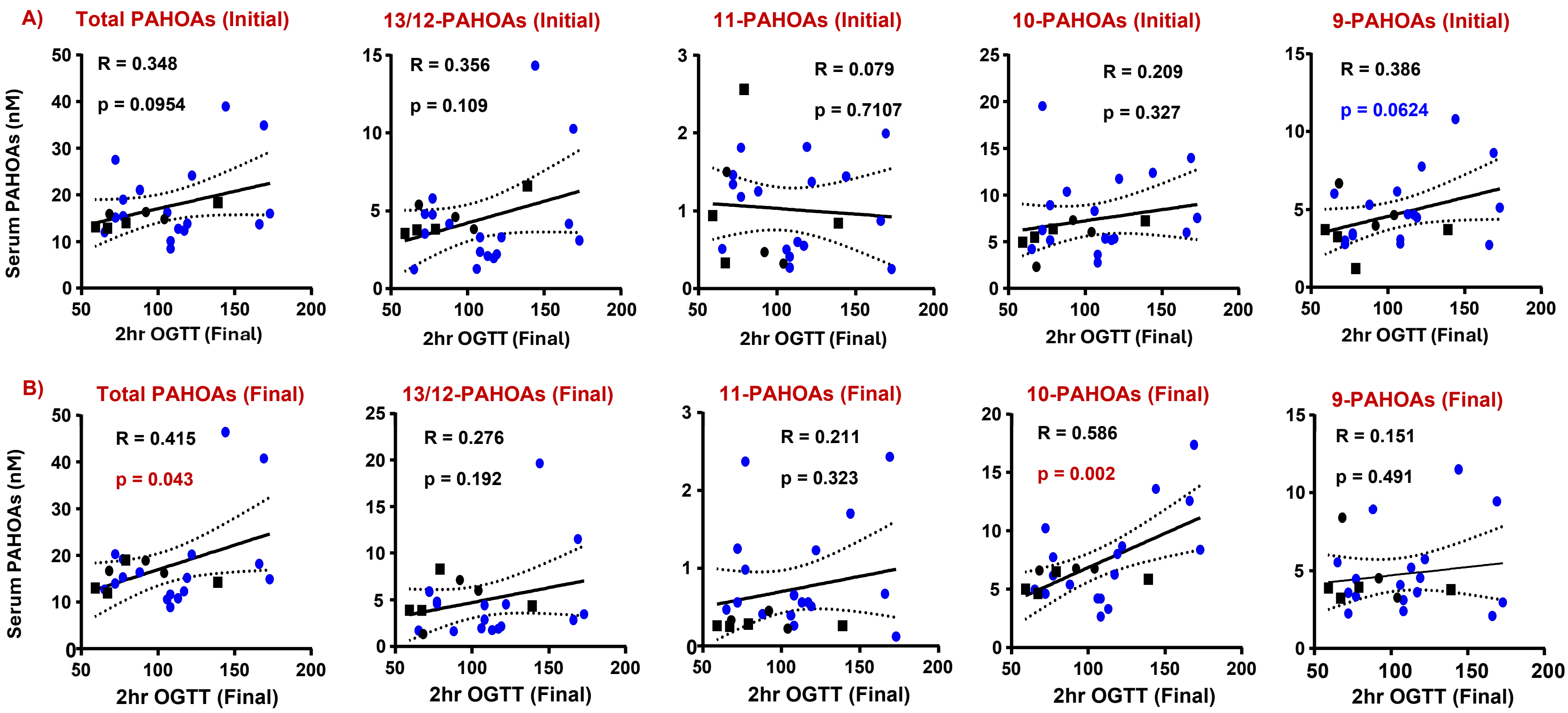

(A) Correlation between initial total PAHOA and individual regioisomers and follow-up 2hr OGTT value in male participants. **(B)** Correlation between final total PAHOA and individual regioisomers and follow-up 2hr OGTT value in male participants. n=24. Correlations were determined by Pearson linear regression analysis. p and R values are on individual graphs. Best fit linear correlation line is shown with 95% Confidence intervals (dotted lines).

Supplementary Figure 9: **Linear correlations between serum PAHOA levels and selected parameters in all participants.**

	OGTT 2hr Glucose values (Final – Initial)	OGTT 2hr Glucose values (Final)	OGTT 2hr Glucose values (Initial)	BMI (Initial)	BMI (Final - Initial)	Age (Initial)	Sex	Follow-up time (Years)	Total PAHOAs (Final)	12/13 - PAHOAs (Final)	11 - PAHOAs (Final)	10 - PAHOAs (Final)	9 - PAHOAs (Final)	Total PAHOAs (Initial)	12/13 - PAHOAs (Initial)	11 - PAHOAs (Initial)	10 - PAHOAs (Initial)	9 - PAHOAs (Initial)	Total PAHOAs (Final - Initial)	12/13 - PAHOAs (Final - Initial)	11 - PAHOAs (Final - Initial)	10 - PAHOAs (Final - Initial)	9 - PAHOAs (Final - Initial)
OGTT 2hr Glucose values (Final – Initial)	1.00	0.78	0.02	0.06	0.32	0.35	-0.27	-0.07	0.34	0.29	0.37	0.35	0.26	0.33	0.27	0.11	0.21	0.38	0.20	0.11	0.35	0.24	-0.20
OGTT 2hr Glucose values (Final)	0.78	1.00	0.65	0.02	0.22	0.49	-0.51	0.08	0.59	0.49	0.55	0.59	0.48	0.55	0.47	0.29	0.39	0.54	0.37	0.17	0.37	0.36	-0.12
OGTT 2hr Glucose values (Initial)	0.02	0.65	1.00	-0.04	-0.04	0.35	-0.49	0.22	0.52	0.43	0.43	0.52	0.45	0.48	0.43	0.33	0.37	0.39	0.34	0.14	0.16	0.28	0.07
BMI (Initial)	0.06	0.02	-0.04	1.00	0.05	0.34	0.12	0.21	0.07	0.16		0.03	0.01	-0.01	0.06	-0.16	-0.10	0.06	0.22	0.20	0.20	0.16	-0.09
BMI (Final - Initial)	0.32	0.22	-0.04	0.05	1.00	0.41	-0.22	0.32	0.06	0.11	0.08	0.04	0.01	0.01	-0.02	-0.10	-0.09	0.16	0.16	0.23	0.22	0.15	-0.24
Age (Initial)	0.35	0.49	0.35	0.34	0.41	1.00	-0.15	0.29	0.47	0.41	0.39	0.43	0.42	0.42	0.37	0.10	0.21	0.54	0.33	0.20	0.39	0.35	-0.20
Sex	-0.27	-0.51	-0.49	0.12	-0.22	-0.15	1.00	-0.28	-0.38	-0.35	-0.40	-0.32	-0.33	-0.39	-0.33	-0.33	-0.31	-0.31	-0.16	-0.14	-0.12	-0.07	-0.02
Follow-up time (Years)	-0.07	0.08	0.22	0.21	0.32	0.29	-0.28	1.00	0.11	0.06	0.13	0.08	0.16	0.05		-0.03	-0.01	0.17	0.17	0.10	0.20	0.12	-0.03
Total PAHOAs (Final)	0.34	0.59	0.52	0.07	0.06	0.47	-0.38	0.11	1.00	0.89	0.89	0.94	0.83	0.94	0.85	0.68	0.73	0.74	0.61	0.34	0.32	0.44	0.10
12/13 - PAHOAs (Final)	0.29	0.49	0.43	0.16	0.11	0.41	-0.35	0.06	0.89	1.00	0.79	0.77	0.58	0.84	0.85	0.63	0.66	0.55	0.54	0.53	0.26	0.28	0.01
11 - PAHOAs (Final)	0.37	0.55	0.43		0.08	0.39	-0.40	0.13	0.89	0.79	1.00	0.82	0.70	0.84	0.79	0.69	0.60	0.68	0.52	0.25	0.46	0.42	-0.01
10 - PAHOAs (Final)	0.35	0.59	0.52	0.03	0.04	0.43	-0.32	0.08	0.94	0.77	0.82	1.00	0.73	0.86	0.75	0.63	0.70	0.67	0.63	0.26	0.31	0.55	0.06
9 - PAHOAs (Final)	0.26	0.48	0.45	0.01	0.01	0.42	-0.33	0.16	0.83	0.58	0.70	0.73	1.00	0.80	0.64	0.54	0.58	0.80	0.45	0.07	0.25	0.32	0.27
Total PAHOAs (Initial)	0.33	0.55	0.48	-0.01	0.01	0.42	-0.39	0.05	0.94	0.84	0.84	0.86	0.80	1.00	0.88	0.72	0.82	0.78	0.30	0.19	0.22	0.21	
12/13 - PAHOAs (Initial)	0.27	0.47	0.43	0.06	-0.02	0.37	-0.33		0.85	0.85	0.79	0.75	0.64	0.88	1.00	0.69	0.58	0.57	0.33	0.01	0.17	0.34	0.07
11 - PAHOAs (Initial)	0.11	0.29	0.33	-0.16	-0.10	0.10	-0.33	-0.03	0.68	0.63	0.69	0.63	0.54	0.72	0.69	1.00	0.54	0.41	0.24	0.08	-0.33	0.22	0.17
10 - PAHOAs (Initial)	0.21	0.39	0.37	-0.10	-0.09	0.21	-0.31	-0.01	0.73	0.66	0.60	0.70	0.58	0.82	0.58	0.54	1.00	0.44	0.13	0.33	0.12	-0.21	0.20
9 - PAHOAs (Initial)	0.38	0.54	0.39	0.06	0.16	0.54	-0.31	0.17	0.74	0.55	0.68	0.67	0.80	0.78	0.57	0.41	0.44	1.00	0.27	0.13	0.38	0.41	-0.36
Total PAHOAs (Final - Initial)	0.20	0.37	0.34	0.22	0.16	0.33	-0.16	0.17	0.61	0.54	0.52	0.63	0.45	0.30	0.33	0.24	0.13	0.27	1.00	0.50	0.39	0.72	0.27
12/13 - PAHOAs (Final - Initial)	0.11	0.17	0.14	0.20	0.23	0.20	-0.14	0.10	0.34	0.53	0.25	0.26	0.07	0.19	0.01	0.08	0.33	0.13	0.50	1.00	0.22	-0.02	-0.10
11 - PAHOAs (Final - Initial)	0.35	0.37	0.16	0.20	0.22	0.39	-0.12	0.20	0.32	0.26	0.46	0.31	0.25	0.22	0.17	-0.33	0.12	0.38	0.39	0.22	1.00	0.28	-0.22
10 - PAHOAs (Final - Initial)	0.24	0.36	0.28	0.16	0.15	0.35	-0.07	0.12	0.44	0.28	0.42	0.55	0.32	0.21	0.34	0.22	-0.21	0.41	0.72	-0.02	0.28	1.00	-0.16
9 - PAHOAs (Final - Initial)	-0.20	-0.12	0.07	-0.09	-0.24	-0.20	-0.02	-0.03	0.10	0.01	-0.01	0.06	0.27		0.07	0.17	0.20	-0.36	0.27	-0.10	-0.22	-0.16	1.00

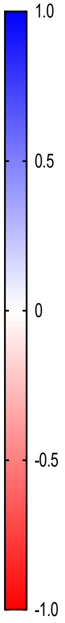

A Pearson linear correlation analysis was performed on the clinical characteristics and serum total PAHOAs and individual regioisomers. The "R" values for the correlations are in the boxes. Collinear Pairs, R > 0.80. The correlation intensity is indicated with red and blue. The darker the blue color, the more positively the parameters correlate with impaired glucose tolerance. The darker the red color, the more negatively the parameters correlate with impaired glucose tolerance.

<u>Supplementary Figure 10</u>: Receiver Operating Characteristic curves and Area Under the Curve (AUC) for initial total PAHOAs and selected derivative parameters.

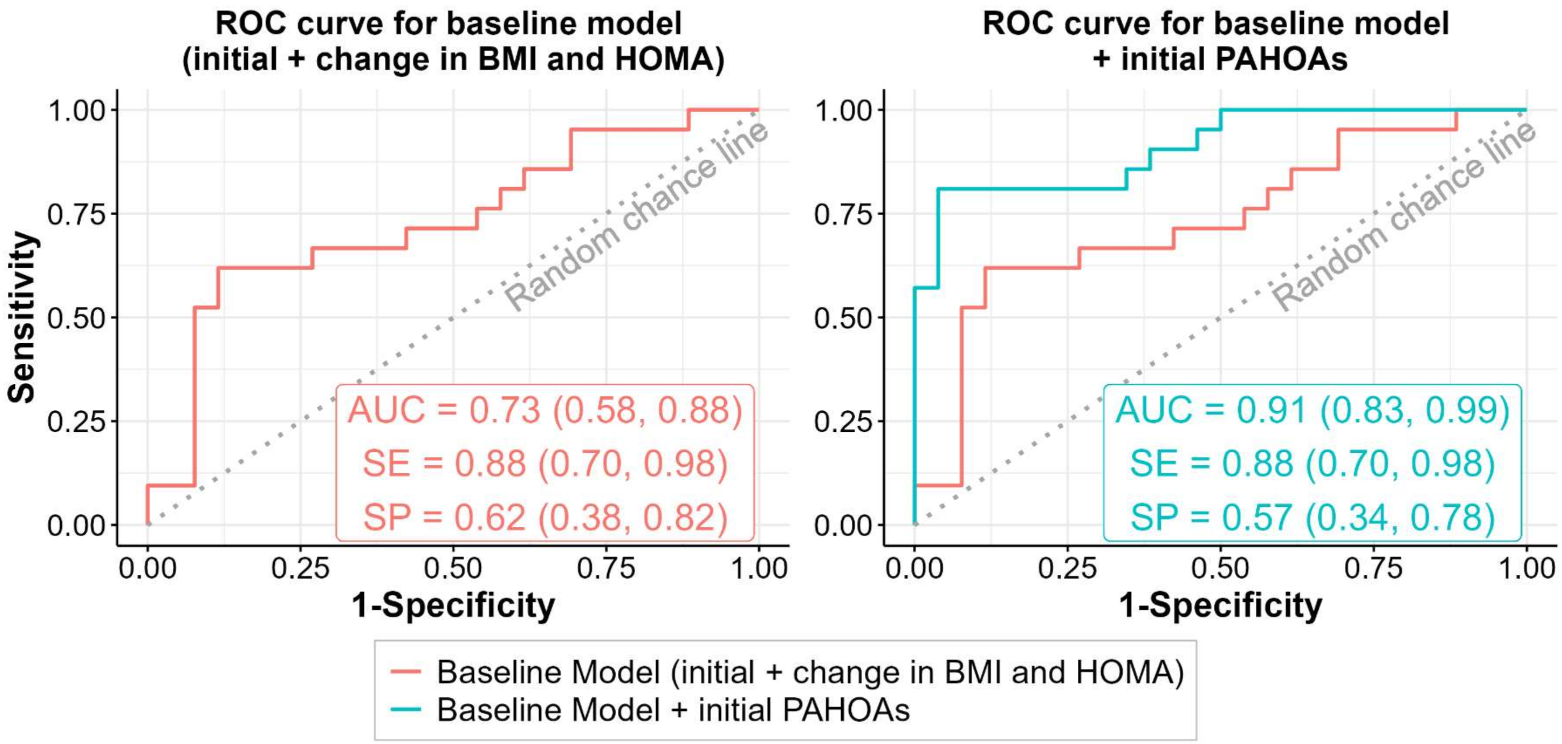

The measures are reported as estimated value (95% Confidence Interval). ROC: Receiver Operating Characteristic; AUC: Area under the Curve; SE: Sensitivity; SP: Specificity; 1-Specificity: False Positive Rate.

Supplementary Table 1: Multivariable Linear Regression Analysis of initial and change in total PAHSAs, 5-PAHSA, 9-PAHSA, initial % body fat, change in % body fat, Age and Sex with change in 2hr-OGTT (final-initial) as outcome variable.

	β (SE)	t	P value	R^2	Adjusted R^2	F (P value)
**Independent Variables**	**Total PAHSAs**			0.37	0.27	3.57 (0.0071)
Initial Total PAHSAs	-9.4865 (3.89)	-2.43	0.0200			
Change in Total PAHSAs	-14.82 (4.54)	-3.26	0.0024			
Sex†: Male	8.31 (10.62)	0.78	0.439			
Age	0.77 (0.55)	1.36	0.1818			
Initial % Body Fat	0.63 (0.63)	1.00	0.3228			
Change in % Body Fat	0.29 (0.96)	0.30	0.7657			
	**5-PAHSA**			0.37	0.27	3.54 (0.0074)
Initial 5-PAHSA	-50.61 (39.29)	-1.28	0.2059			
Change in 5-PAHSA	-128.35 (37.6)	-3.41	0.0016			
Sex†: Male	6.89 (10.78)	0.64	0.5268			
Age	0.39 (0.54)	0.71	0.4808			
Initial % Body Fat	0.63 (0.63)	0.99	0.3275			
Change in % Body Fat	0.68 (0.95)	0.71	0.4791			
	**9-PAHSA**			0.30	0.19	2.59 (0.0342)
Initial 9-PAHSA	-15.78 (6.84)	-2.31	0.0268			
Change in 9-PAHSA	-17.89 (8.00)	-2.23	0.0317			
Sex†: Male	7.57 (11.41)	0.66	0.511			
Age	0.96 (0.56)	1.71	0.0958			
Initial % Body Fat	0.66 (0.66)	0.99	0.3268			
Change in % Body Fat	0.17 (1.01)	0.17	0.8652			

Analysis was performed in male and female participants combined. β Estimate: Standardized Coefficient Beta; SE: Standard Error; F: F-statistic; t: t-test statistic; † Female is the reference category in the multivariable model.

Supplementary Table 2: Multivariable Linear Regression Analysis of initial and change in total PAHSAs, initial fasting insulin, initial fasting glucose, and initial triglyceride/HDL with change in 2hr-OGTT (final-initial) as outcome variable.

A)

	**β (SE)**	**t**	**P value**	**R^2**	**Adjusted R^2**	**F (P value)**
Independent Variables	**Change in 2hr-OGTT Value**			0.31	0.26	6.61 (0.0009)
Initial total PAHSAs	-7.21 (3.52)	-2.05	0.0465			
Change in total PAHSAs	-17.62 (4.03)	-4.37	0.0001			
Initial fasting insulin	-0.97 (1.04)	-0.94	0.354			

B)

	**β (SE)**	**t**	**P value**	**R^2**	**Adjusted R^2**	**F (P value)**
Independent Variables	**Change in 2hr-OGTT Value**			0.30	0.25	6.27 (0.0012)
Initial total PAHSAs	-7.80 (3.68)	-2.12	0.0395			
Change in total PAHSAs	-17.88 (4.19)	-4.27	0.0001			
Initial fasting glucose	2.99 (7.69)	0.39	0.6989			

C)

	**β (SE)**	**t**	**P value**	**R^2**	**Adjusted R^2**	**F (P value)**
Independent Variables	**Change in 2hr-OGTT Value**			0.30	0.25	6.14 (0.0014)
Initial Total PAHSAs	-7.71 (3.64)	-2.11	0.0398			
Change in Total PAHSAs	-17.35 (4.10)	-4.22	<0.0001			
Initial Triglyceride/HDL ratio	4.39 (7.87)	0.54	0.5796			

Analysis was performed in male and female participants combined. β Estimate: Standardized Coefficient Beta; SE: Standard Error; F: F-statistic; t: t-test statistic.

Supplementary Table 3: Multivariable Linear Regression Analysis of initial and change in 5-PAHSA and 9-PAHSA and initial triglyceride/HDL with change in 2hr-OGTT (final-initial) as outcome variable.

A)

	**β (SE)**	**t**	**P value**	**R^2**	**Adjusted R^2**	**F (P value)**
Independent Variables	**Change in 2hr-OGTT Value**			0.34	0.29	7.25 (0.0005)
Initial 5-PAHSA	-70.98 (35.9)	-1.94	0.0544			
Change 5-PAHSA	-149.7 (33.7)	-4.38	<0.0001			
Initial Triglyceride/HDL ratio	6.85 (7.60)	0.88	0.3727			

B)

	**β (SE)**	**t**	**P value**	**R^2**	**Adjusted R^2**	**F (P value)**
Independent Variables	**Change in 2hr-OGTT Value**			0.19	0.14	3.44 (0.0250)
Initial 9-PAHSA	-12.37 (6.75)	-1.83	0.0737			
Change in 9-PAHSA	-22.50 (7.30)	-3.08	0.0036			
Initial Triglyceride/HDL ratio	6.81 (8.76)	0.78	0.4412			

Analysis was performed in male and female participants combined. β Estimate: Standardized Coefficient Beta; SE: Standard Error; F: F-statistic; t: t-test statistic.

Supplementary Table 4: Multivariable Linear Regression Analysis of initial and change in total PAHSAs, 5-PAHSA, 9-PAHSA, initial BMI, change in BMI, Age and Sex with change in final 2hr-OGTT as outcome variable.

	B (SE)	B*	P value	R^2	Adjusted R^2	F (P value)
Independent Variables	**OGTT at baseline**			0.45	0.39	8.48 (<0.0001)
Sex†: Male	-30.51 (8.47)	-30.51	0.0008			
Age	2.26 (0.63)	17.33	0.0008			
Initial BMI	-0.86 (1.43)	-2.64	0.5516			
Change in BMI	-1.47 (2.65)	-2.53	0.5818			
	**Total PAHSAs**			0.49	0.41	6.47 (<0.0001)
Initial Total PAHSAs	-5.83 (4.57)	-6.14	0.2094			
Change in Total PAHSAs	-9.25 (5.20)	-8.52	0.0831			
Sex†: Male	-24.82 (8.84)	-24.82	0.0077			
Age	2.19 (0.67)	16.80	0.0022			
Initial BMI	-0.79 (1.41)	-2.43	0.5802			
Change in BMI	-1.60 (2.67)	-2.75	0.5531			
	**5-PAHSA**			0.60	0.54	9.96 (<0.0001)
Initial 5-PAHSA	-100.11 (41.03)	-9.56	0.0192			
Change in 5-PAHSA	-137.64 (39.91)	-13.83	0.0013			
Sex†: Male	-19.91 (7.95)	-19.91	0.0165			
Age	1.63 (0.59)	12.51	0.0082			
Initial BMI	-0.30 (1.26)	-0.91	0.8149			
Change in BMI	-0.07 (2.42)	-0.11	0.9787			
	**9-PAHSA**			0.50	0.43	6.74 (<0.0001)
Initial 9-PAHSA	-6.98 (7.29)	-4.39	0.3443			
Change in 9-PAHSA	-18.27 (8.62)	-10.01	0.0403			
Sex†: Male	-23.41 (8.90)	-23.41	0.0121			
Age	2.14 (0.63)	16.37	0.0016			
Initial BMI	-0.82 (1.40)	-2.53	0.5608			
Change in BMI	-1.42 (2.59)	-2.44	0.5877			

Analysis was performed in male and female participants combined. β Estimate: Standardized Coefficient Beta; SE: Standard Error; F: F-statistic; t: t-test statistic; † Female is the reference category in the multivariable model.

Supplementary Table 5: Multivariable Linear Analysis of initial total PAHSAs, change in total PAHSAs and follow-up time with change in 2hr-OGTT value (Final – Initial) or Final 2hr-OGTT value being the dependent variable.

A)

	**β (SE)**	**t**	**P value**	**R^2**	**Adjusted R^2**	**F (P value)**
Independent Variables	**Change in 2hr-OGTT**			0.30	0.25	6.34 (0.0011)
Initial Total PAHSAs	-7.41 (3.55)	2.09	0.0426			
Change in Total PAHSAs (Final-Initial)	-17.49 (4.06)	4.31	<0.0001			
Follow-up time in Years	-0.56 (1.20)	1.2	0.6425			

B)

	**β (SE)**	**t**	**P value**	**R^2**	**Adjusted R^2**	**F (P value)**
Independent Variables	**Final 2hr-OGTT**			0.21	0.16	3.88 (0.0152)
Initial Total PAHSAs	-5.63 (4.96)	1.93	0.2627			
Change in Total PAHSAs (Final-Initial)	-19.01 (5.68)	3.42	<0.0001			
Follow-up time in Years	1.13 (1.68)	0.19	0.5048			

Analysis was performed in male and female participants combined. β Estimate: Standardized Coefficient Beta; SE: Standard Error; F: F-statistic; t: t-test statistic.

Supplementary Table 6: Pearson correlations for serum PAHSA levels with Final 2hr-OGTT.

**Correlation with Final 2hr OGTT Value**	**Pearson R**	**p-value**
Initial Total PAHSAs	-0.395	0.0027
Initial 5-PAHSA	-0.303	0.0183
Change in Total PAHSA (Final-Initial)	-0.4766	0.0003
Change in 5-PAHSA (Final-Initial)	-0.5058	0.0001
Final 5-PAHSA	-0.50	0.0001
Initial % Body Fat	0.2564	0.0446

Supplementary Table 7: Pearson correlations for serum PAHOA levels with Final 2hr-OGTT.

Serum PAHOA levels	Pearson R	p-Value	Confidence interval of Pearson R
10-PAHOA (Final)	0.5948	0.00000825	0.3739 to 0.7519
Total PAHOA (Final)	0.5896	0.00001034	0.3670 to 0.7484
11- PAHOA (Final)	0.5501	0.00005113	0.3152 to 0.7215
Total PAHOA (Initial)	0.5488	0.00005374	0.3135 to 0.7206
9-PAHOA (Initial)	0.5353	0.00008858	0.2962 to 0.7112
13/12-PAHOA (Final)	0.4903	0.00040392	0.2395 to 0.6797
9-PAHOA (Final)	0.4775	0.00059981	0.2237 to 0.6706
13/12-PAHOA (Initial)	0.4728	0.00068964	0.2180 to 0.6673
10-PAHOA (Initial)	0.3912	0.00597266	0.1204 to 0.6078
Total PAHOA (Final-Initial)	0.3690	0.00985576	0.09480 to 0.5911
11-PAHOA (Final-Initial)	0.3660	0.01051764	0.09137 to 0.5889
10-PAHOA (Final-Initial)	0.3585	0.0123	0.08285 to 0.5833
11-PAHOA (Initial)	0.2874	0.0476	0.003517 to 0.5284
13/12-PAHOA (Final-Initial)	0.1730	0.2395	-0.1169 to 0.4357
9-PAHOA (Final-Initial)	-0.1165	0.4304	-0.3878 to 0.1734

<u>Supplementary Table 8</u>: Multivariable Linear Regression Analysis of initial total PAHOAs, initial % body fat, change in % body fat, Age and Sex with final 2hr-OGTT value as outcome variable.

	**β (SE)**	**B***	**P value**	**R^2**	**Adjusted R^2**	**F (P value)**
Independent Variables	**Final 2hr-OGTT Value**			0.49	0.43	7.41 (<0.0001)
Initial PAHOA	1.02 (0.48)	10.01	0.0389			
Initial % Body Fat	0.31 (0.70)	2.52	0.6648			
Change in % Body Fat	-0.16 (0.87)	-1.49	0.8527			
Age	1.37 (0.61)	10.49	0.0311			
Sex†: Male	-20.96 (11.93)	-20.96	0.0871			

Analysis was performed in male and female participants combined. β Estimate: Standardized Coefficient Beta; SE: Standard Error; F: F-statistic; t: t-test statistic; † Female is the reference category in the multivariable model.

<u>Supplementary Table 9</u>: Multivariable Linear Analysis in all participants of initial serum total PAHOA levels, initial fasting insulin, initial fasting glucose, and initial triglyceride/HDL with final 2hr-OGTT value as outcome variable.

A)

	**β (SE)**	**t**	**P value**	**R^2**	**Adjusted R^2**	**F (P value)**
Independent Variables	**Final 2hr-OGTT Value**			0.30	0.27	9.83 (0.0003)
Initial PAHOA	1.97 (0.46)	4.23	<0.0001			
Initial fasting Insulin	-0.30 (1.40)	-0.22	0.8313			

B)

	**β (SE)**	**t**	**P value**	**R^2**	**Adjusted R^2**	**F (P value)**
Independent Variables	**Final 2hr-OGTT Value**			0.32	0.29	10.82 (0.0001)
Initial PAHOA	2.03 (0.44)	4.55	<0.0001			
Initial fasting Glucose	11.21 (9.39)	1.19	0.2388			

C)

	**β (SE)**	**t**	**P value**	**R^2**	**Adjusted R^2**	**F (P value)**
Independent Variables	**Final 2hr-OGTT Value**			0.30	0.27	8.51 (0.0004)
Initial PAHOA	2.02 (0.46)	4.43	<0.0001			
Initial Triglyceride/HDL ratio	3.29 (10.04)	0.32	0.7446			

Analysis was performed in male and female participants combined. β Estimate: Standardized Coefficient Beta; SE: Standard Error; F: F-statistic; t: t-test statistic.
